# Variations in Innate Immune Cell Subtypes Correlate with Epigenetic Clocks, Inflammaging and Health Outcomes

**DOI:** 10.1002/advs.202505922

**Published:** 2025-08-27

**Authors:** Xiaolong Guo, Josephine A. Robertson, Andrea Aparicio, Kirsten Seale, Qingwen Chen, Anne Richmond, Zhaozhen Du, Mahnoor Sulaiman, Shijie C Zheng, Esteban Ballestar, Charlotte AM Cecil, Bastiaan T Heijmans, Steve Horvath, Varun B. Dwaraka, Jessica Lasky‐Su, Ryan Smith, Riccardo E. Marioni, Andrew E Teschendorff

**Affiliations:** ^1^ Shanghai Institute of Nutrition and Health University of Chinese Academy of Sciences Chinese Academy of Sciences 320 Yue Yang Road Shanghai 200031 China; ^2^ Centre for Genomic and Experimental Medicine Institute of Genetics and Cancer University of Edinburgh Edinburgh EH4 2XU UK; ^3^ Channing Division of Network Medicine Department of Medicine Brigham and Women's Hospital and Harvard Medical School Boston MA 02115 USA; ^4^ TruDiagnostics 881 Corporate Dr Lexington KY 40503 USA; ^5^ Department of Biomedical Data Sciences Leiden University Medical Center Einthovenweg 20 Leiden 2333 ZC The Netherlands; ^6^ Department of Child and Adolescent Psychiatry/Psychology Sophia's Children Centre Erasmus MC Rotterdam 3015 CN The Netherlands; ^7^ Pfizer Research & Development, Pfizer Inc. Groton Connecticut CT06340 USA; ^8^ Epigenetics and Immune Disease Group Josep Carreras Leukaemia Research Institute (IJC) Badalona Barcelona 08916 Spain; ^9^ Epigenetics in Inflammatory and Metabolic Diseases Laboratory Health Science Center (HSC) East China Normal University (ECNU) Shanghai 200241 China; ^10^ Altos Labs Cambridge CB21 6GP UK

**Keywords:** aging Biomarkers, DNA methylation, epigenetic clocks, health outcomes, innate immune system, inflammaging

## Abstract

Epigenetic clocks in blood have shown promise as tools to quantify biological age, displaying robust associations with morbidity and all‐cause mortality. Whilst the effect of cell‐type heterogeneity on epigenetic clock estimates has been explored, such studies have been limited to studying heterogeneity within the adaptive immune system. Much less is known about whether heterogeneity within the innate immune system can impact epigenetic clock estimates and their associations with health outcomes. Here, we apply a high‐resolution DNAm reference panel of 19 immune cell‐types, including young and adult monocyte, natural killer, and neutrophil subsets, demonstrating how shifts within these innate subtypes display associations with epigenetic clock acceleration, inflammaging, and all‐cause mortality. The associations of monocyte heterogeneity with inflammation are further validated using transcriptomic and metabolomic data. Additionally, a non‐negligible fraction of nucleated red blood cell‐like cells in circulation is found to associate with inflammaging, markers of dysfunctional erythropoiesis, and is a major risk factor for all‐cause mortality. These results extend findings obtained within the adaptive immune system to innate immune and erythrocyte‐like cells, demonstrating how heterogeneity within these other blood cell compartments is also associated with inflammaging, epigenetic clocks, and health outcomes.

## Graphical abstract

This study uses a high‐resolution DNA methylation reference panel for 19 immune cell‐types, as well as transcriptomic, metabolomic, and large population cohort data to show that age‐related epigenetic heterogeneity within monocytes correlates with inflammaging, biological age, and all‐causemortality, and does so more strongly than existing monocyte classifications. The same panel also identifies an erythroblast‐like component that correlates with dysfunctional erythropoiesis and all‐cause mortality. This heterogeneity drives another component of epigenetic clocks, yet biological aging clocks remain independently associated with cause‐specific and all‐cause mortality.

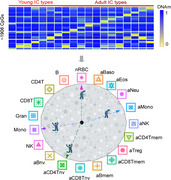

## 1 Introduction

Epigenetic clocks are promising tools for predicting chronological and biological age from easily accessible tissues like blood.^[^ ^1^, ^2^, ^3^, ^4^ ^]^ Of particular interest are their associations with health outcomes, such as all‐cause mortality.^[^ ^4^, ^5^, ^6^ ^]^ However, a full elucidation of the biological processes driving these associations is still ongoing.^[^ ^3^, ^7^, ^8^ ^]^ Because most epigenetic clocks have been built from a heterogeneous mixture like blood^[^ ^5^, ^9^, ^10^, ^11^, ^12^ ^]^ (or at least predominantly so^[^ ^13^ ^]^), one biological process known to contribute to epigenetic clock estimates is shifts in the underlying immune cell‐type proportions.^[^ ^3^, ^14^, ^15^ ^]^ This has recently been quantified in relation to chronological age prediction, with up to 40% of an epigenetic clock's accuracy in blood being driven by age‐related shifts in lymphocyte subsets, specifically by an age‐associated increase in the memory to naïve T‐cell ratio.^[^ ^16^ ^]^ Moreover, this shift between naïve and memory T‐cell fractions can drive biological age estimates,^[^ ^17^ ^]^ with the naïve CD4+ T‐cell fraction in particular being protective against all‐cause mortality.^[^ ^18^ ^]^ Reflecting these age‐related shifts between memory and naïve T‐cell fractions are known age‐associated processes such as immune‐senescence and chronic low‐grade inflammation, termed “inflammaging.”^[^ ^19^, ^20^ ^]^ Thus, while it is clear that a component of epigenetic clocks is driven by “inflammaging,” their associations with chronological and biological age are also independent of it, suggesting that other biological mechanisms or effects may be contributing to the associations with quantifiable biological age measures like all‐cause mortality.

Here, we explore the possibility that shifts in cell‐type heterogeneity within other immune‐cell compartments, notably the innate immune system, which encompasses granulocytes, monocytes, and natural killer cells, also contribute to epigenetic clock estimates and the ensuing associations with health outcomes. However, to explore this question would require a priori specification of relevant cell subtypes within each of these major innate immune cell‐types. This is challenging as these subtypes are not well‐characterized, and DNA methylation (DNAm) profiles for corresponding subtypes have not yet been generated, except arguably for monocytes.^[^ ^21^, ^22^, ^23^ ^]^ Nevertheless, we reasoned that innate immune cell types could be subtyped in relation to how “young” or “old” they appear in a given blood sample, irrespective of the chronological age of the donor. In particular, we hypothesized that cord and adult blood innate immune cell‐types, which have been abundantly profiled at the DNAm‐level,^[^ ^24^ ^]^ could be used as “yardsticks” to quantify the relative proportions of “young” and “old” innate immune cell‐types in any blood sample, including those from adult cohorts. Supporting this, we recently generated from sorted cord and adult blood DNAm datasets, a 19 immune‐cell type DNAm reference panel called UniLIFE, which we have extensively validated in infants, children, adolescents, and adults.^[^ ^24^ ^]^ Here, we adapt UniLIFE to adult cohorts only, to explore if the underlying estimates of young and old immune cell‐types are informative of chronological and biological age. We find that many shifts between the young and old innate immune‐cell type fractions correlate with epigenetic clocks and health outcomes, including notably all‐cause mortality. In particular, we show that a shift in the young to old monocyte fraction correlates with inflammaging and with all‐cause mortality, independently of major disease risk factors. This association with inflammaging is validated in a cohort with matched metabolomic data and is stronger than that displayed by traditional monocyte subtyping into classical and non‐classical ones. In addition, we find that the fraction of a rare cell‐type, possibly reflecting circulating nucleated red blood cells (nRBCs) or erythroblasts in adult blood, increases with age, whilst also increasing the risk of all‐cause mortality independently of age and other risk factors. Validating this association, we find that the nRBC fraction correlates with markers of dysfunctional erythropoiesis. This points toward a form of aberrant erythropoiesis as a previously unrecognized contributor to the epigenetic clock's associations with health outcomes.

## 2 Results

### 2.1 A common Axis of DNAm Variation Distinguishes Cell‐Types by Maturity and Age

**Figure 1:**
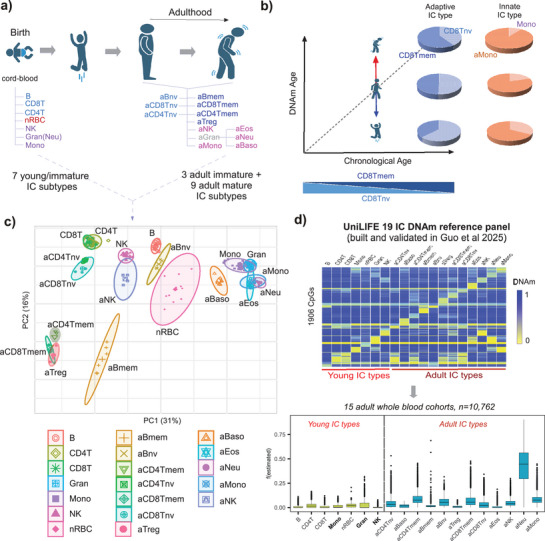
A common axis of DNAm variation reflects maturity and aging across adaptive and innate immune cell‐types. a) Sorting and DNAm profiling of 7 immune cell‐types found in cord‐blood (birth) and 12 immune cell‐types found in adult blood (prefixed with “a”) could be used to identify shifts of “young” and “old” cellular phenotypes during adulthood, within both adaptive and innate immune cell‐types. b) Conceptualization of our hypothesis: individuals aging faster than normal (according to some epigenetic clock) could be driven in part by a subtle shift in the balance between “young” and “old” immune cell subtypes. In the case of an adaptive immune cell‐type, it is well‐known that the adult naïve CD8+ T‐cell fraction decreases with age at the expense of an increase in the adult memory CD8+ T‐cell fraction, and that further tilting of this balance at any age indicates DNAm age acceleration or deceleration, as shown. In the case of an innate immune cell‐type, e.g. monocytes, we hypothesize that the balance between “young” and “old” subtypes could also associate with DNAm age acceleration, for instance, because the younger and older monocyte subtypes reflect non‐inflammatory, respectively, inflammatory subtypes. c) PCA plot of a merged Illumina 450k DNAm cohort of over 220 sorted samples, encompassing the 19 immune cell‐types depicted in a). It is observed how PC2 describes an axis of DNAm variation that correlates simultaneously with maturity and age, and that is common to all adaptive and innate immune cell‐types. PC1 describes an axis of DNAm variation associated with lymphoid versus myeloid status. d) Using the merged DNAm cohort, a 19 immune cell‐type UniLIFE DNAm reference panel was built. Details of its construction and validation are elaborated in Guo et al. The UniLIFE reference panel was applied to a collection of 15 adult whole blood cohorts to estimate corresponding fractions for the 19 immune cell‐types, as shown in the boxplots. Parts of panel a)+b) were generated with Biorender.com.

We hypothesized that cord and adult peripheral immune cell‐types, including innate types such as neutrophils or monocytes, could be utilized as “yardsticks” in a continuum that reflect variable proportions of “younger” and “older” cell phenotypes, and that such variations in the corresponding cell‐type fractions could be informative of biological age (**Figure** **1**a,b). Justifying this hypothesis, we note that a PCA‐analysis of an Illumina 450k DNAm dataset representing a merged cohort of over 200 sorted samples encompassing 19 cord and adult immune cell‐types, demonstrates how adult naïve adaptive (CD4+ and CD8+ T‐cells, B‐cells) and adult innate immune cell‐types (natural killer cells, monocytes and neutrophils) are all distinguishable from their cord‐blood counterparts along the same axis of variation (Figure 1c).^[^ ^24^ ^]^ Importantly, adult memory lymphocytes were more separated from their adult naïve counterparts, indicating that for lymphocytes, adult naïve cell‐types are more similar to their cord‐blood counterparts, reflecting their joint inexperience to antigen‐exposure (Figure 1c). Thus, the same axis of variation can discriminate adult cell‐types according to their level of antigen‐exposure (e.g., adult memory CD4+ T‐cells from adult naïve CD4+ T‐cells), as well as according to their age (e.g., adult naïve CD4+ T‐cells from cord‐blood (naïve) CD4+ T‐cells, or adult monocytes from cord‐blood monocytes). From this merged cohort, we previously derived and validated a 19 immune cell‐type DNAm reference panel called UniLIFE (Figure 1d),^[^ ^24^ ^]^ allowing estimation of corresponding immune cell fractions in any blood sample. Hence, to test our hypothesis (Figure 1b), we here applied UniLIFE to 15 adult whole blood cohorts encompassing over 10 000 blood samples^[^ ^18^ ^]^ (Methods), estimating fractions for 7 “young” (i.e., cord‐blood) and 12 adult blood cell‐types (Figure 1d). Whilst young (i.e, cord‐blood) fractions were, as required, significantly lower than corresponding adult fractions (Figure 1d, Figure S1, Supporting Information) (also validated by us previously^[^ ^24^ ^]^), these young cell fractions were non‐negligible (Figure S2a, Supporting Information). Attesting to the quality of our UniLIFE panel, the estimated nucleated red blood cell (nRBC) fraction was much higher in cord‐blood samples and independently sorted nRBC samples compared to the adult cohorts (Figure S2b, Supporting Information).^[^ ^24^ ^]^ We reasoned that a given non‐zero young cell fraction in adult blood could mark an adult individual whose corresponding blood cell‐type appears on average “younger” than expected, for instance, because of a larger subpopulation of cells that retain features of their cord‐blood counterpart. This would be of particular novel interest for innate immune cell‐types like neutrophils, natural killer (NK) cells, or monocytes, since for adaptive immune cell‐types (e.g., CD4+ and CD8+ T‐cells, B‐cells) the well‐characterized adult naïve compartment is already representative of a younger cell phenotype (Figure 1c). In other words, for innate immune cell‐types such as neutrophils, monocytes, and NK‐cells, a corresponding non‐zero “cord‐blood” fraction in an adult blood sample could reflect a relatively higher proportion of younger cells, which could be of biological and clinical significance.

### 2.2 Shifts Within Innate Immune Cell‐Types Associate with Chronological Age and Sex

To begin, we first tested for associations of the 19 immune cell fractions with age and sex. Performing a meta‐analysis over the 15 adult blood cohorts (Table S1, Supporting Information), whilst adjusting for sex, we observed patterns of association with age (Figure S3, Supporting Information) that were strongly consistent with our earlier study performed at the resolution of 12 adult immune cell‐types.^[^ ^18^ ^]^ For instance, adult naïve B‐cell, CD8+, and CD4+ T‐cell fractions decrease with age, whilst corresponding memory T‐cell fractions increase (Figure S3, Supporting Information). Although fractions for the young cell‐types were small, these fractions decreased further with age (Figure S3, Supporting Information). Patterns of association with sex, whilst adjusting for age, were broadly speaking also consistent with our previous results inferred at the resolution of 12 immune cell‐types (Figure S4, Supporting Information).^[^ ^18^ ^]^ For instance, naïve CD4+ T‐cell and B‐cell fractions were higher in women compared to men. Interestingly, in women, the young and adult monocyte fractions were significantly higher and lower, respectively, compared to men (Figure S4, Supporting Information).

### 2.3 Shifts Within Innate Immune Cell‐Types Associate with Epigenetic Clock Acceleration

**Figure 2:**
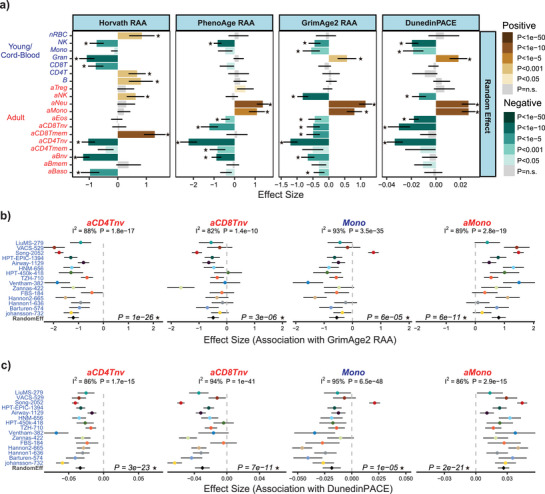
Shifts within innate immune cell‐types correlate with age‐acceleration. a) For each of the 19 immune cell‐types in the UniLIFE DNAm reference panel, we give the effect sizes of association of the cell‐type fraction with the relative age‐acceleration of Horvath, PhenoAge, and GrimAge2 clocks, as well as with DunedinPACE's pace of aging, for a random effect meta‐analysis model, as assessed over 15 adult whole blood cohorts. Error bars represent 95% confidence intervals. b) For GrimAge2, detailed forest plots for 4 of the cell‐type fractions across the 15 adult blood cohorts: adult naïve CD4+ and CD8+ T‐cells (aCD4Tnv, aCD8Tnv), young (cord‐blood) monocytes (Mono), and adult monocytes (aMono). P‐values for the random effect model are given. Heterogeneity I^2^ indices and P‐values are given above each panel. c) As b) but for DunedinPACE. *Significant under Bonferroni correction.

To explore our hypothesis that the young and old innate immune cell‐types may be of biological significance, we next computed the relative age acceleration (RAA, also known as “extrinsic age acceleration”, EAA, Methods) of various epigenetic clocks, including Horvath,^[^ ^13^ ^]^ PhenoAge,^[^ ^10^ ^]^ GrimAge2^[^ ^5^ ^]^ and DunedinPACE,^[^ ^12^ ^]^ in each of the 15 adult blood cohorts, to see if any of the 19 immune cell fractions associate with RAA (**Figure** **2**a). As expected, for the Horvath clock, RAA was strongly associated with an increased memory CD8+ T‐cell and decreased naïve CD4+ T‐cell fractions (Figure 2a, Figure S5, Supporting Information), indicating how shifts within these T‐cell compartments can drive RAA, consistent with previous reports.^[^ ^17^ ^]^ In contrast, PhenoAge RAA displayed a much stronger inverse association with naïve fractions in all adaptive lymphocyte subsets, including CD4+ T‐cells, CD8+ T‐cells, and B‐cells, whilst the adult neutrophil fraction correlated positively (Figure 2a, Figure S6, Supporting Information), patterns that are broadly consistent with the original PhenoAge clock publication.^[^ ^10^ ^]^ GrimAge2 and DunedinPACE displayed similar patterns of association, with increased adult naïve CD4+ and CD8+ T‐cell fractions reducing GrimAge2‐RAA and the pace of aging (Figure 2a–c, Figures S7 and S8, Supporting Information). However, we also observed strong associations of DunedinPACE and GrimAge2 RAA with innate immune cell‐type fractions (Figure 2a–c, Figures S7 and S8, Supporting Information). Specifically, an increased young monocyte fraction also reduced the pace of aging and RAA, whilst the old monocyte fraction correlated positively (Figure 2b,c, Figures S7 and S8, Supporting Information). This suggests that current epigenetic clocks for biological age are also influenced by heterogeneity within innate immune cell‐types.

### 2.4 Differential Young Versus Adult Monocyte Fraction Associates with Inflammaging

**Figure 3:**
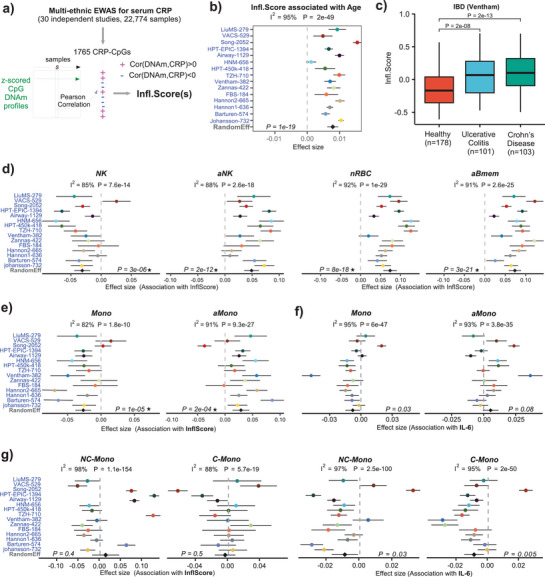
Elevated adult to young monocyte fractions correlate with inflammaging. a) Flowchart depicting the calculation of the DNAm‐based inflammaging score (Infl.Score) for a sample s. A large EWAS meta‐analysis of measured C‐reactive protein (CRP) levels in serum identified a total of 1765 CRP‐associated CpGs. For an independent DNAm dataset, we first z‐score the CpG DNAm profiles across all samples in the dataset, so that the mean is zero and the standard deviation of each CpG is 1. The InflScore of sample s is then defined as the Pearson correlation of z‐score normalized values with the bidirectional vector of the 1765 CpGs, with +1/‐1 indicating positive/negative correlation with CRP. b) Validation forest plot of the InflScore against chronological age, as assessed over 15 adult whole blood cohorts. *P*‐value of the random effect model is given. Above the panel, we list the I^2^ heterogeneity index and associated P‐value. c) Boxplots of the InflScore in an EWAS of inflammatory bowel disease, comparing ulcerative colitis and Crohn's Disease cases to healthy controls. *P*‐values are from a one‐tailed Wilcoxon rank sum test. d) Forest plots of association of young (NK) and adult (aNK) natural killer fractions, nRBC, and adult memory B‐cell fractions with the DNAm‐based CRP Infl.Score. P‐values as in b). e) As d), but for young (Mono) and adult (aMono) monocyte fractions. f) As e), but the association with the DNAm‐based IL6 score. g) As e+f, but for estimated classical (C‐Mono) and non‐classical (NC‐Mono) fractions. *Significant under Bonferroni correction.

That the young and adult monocyte fractions displayed negative, respectively positive, associations with the pace of aging and GrimAge2/PhenoAge's RAA across so many independent cohorts, suggests that this heterogeneity within monocytes could be biologically significant. In particular, we hypothesized that the relative proportions of young and old monocytes in blood could reflect the proportion of a known (or unknown) inflammatory monocyte subtype associated with a condition such as low‐grade chronic inflammation (“inflammaging”). To test this, we first built an inflammaging score from a large multi‐ethnic meta‐analysis of EWASs, where DNAm was correlated to measured serum levels of C‐reactive protein (CRP), a well‐known marker of inflammation, including low‐grade chronic inflammation (**Figure** **3**a, Methods).^[^ ^25^ ^]^ This meta‐analysis identified, with high confidence, a total of 1765 CpGs whose DNAm levels are correlated with CRP, as assessed over 22774 whole blood samples from 30 independent studies. Restricting to these 1765 CpGs, the sample‐specific inflammation score (InflScore) was then computed as the Pearson correlation of a sample's z‐score normalized DNAm profile with the corresponding bi‐directional vector specifying the directionality of change of the 1765 CpGs in relation to CRP levels (Figure 3a, Methods). Consistent with this score measuring inflammaging, we found it to be strongly correlated with chronological age across all cohorts (Figure 3b). It was also increased in hallmark “inflammaging” diseases such as ulcerative colitis or Crohn's disease (Figure 3c). When calculated in the 15 adult whole blood cohorts, we also verified that InflScore captures the increase in memory CD4+ T‐cell fractions compared to naïve ones (Figure S9, Supporting Information). A similar pattern was also observed for natural‐killer cells, with the young and adult subtypes correlating negatively and positively with the inflammaging score (Figure 3d). Consistent with antigen exposure being associated with inflammation, the adult memory B‐cell fraction also displayed strong positive associations with the InflScore (Figure 3d). Importantly, the young and adult monocyte fractions correlated negatively and positively with the CRP‐derived inflammaging score (Figure 3e), thus demonstrating that these shifts within the monocyte subset are of biological significance. To further confirm these associations with inflammaging, we repeated the analysis for a DNAm‐based score of IL‐6 levels^[^ ^26^, ^27^ ^]^ (Methods). This IL‐6 score also displayed strong associations with age in every cohort examined (Figure S10a, Supporting Information), and with inflammatory conditions like RA and IBD (Figure S10b, Supporting Information). Meta‐analysis over our 15 cohorts further revealed similar associations with the 19 immune cell fractions as seen for our CRP‐based InflScore (Figure S10c, Supporting Information). For instance, the IL‐6 score correlated positively and negatively with the adult and young monocyte fractions, respectively (Figure 3f, Figure S10d, Supporting Information). Overall, these data support the view that a shift between the adult and young monocyte fraction is a marker of inflammaging.

### 2.5 Monocyte Subtyping into Young and Old Phenotypes is Distinct From Classical Subtyping

The observed heterogeneity within monocytes could, in principle, also reflect variations in the classical versus non‐classical monocyte subtypes.^[^ ^28^ ^]^ To explore this possibility, we processed an Illumina 450k DNAm dataset of sorted classical and non‐classical monocytes from healthy and systemic lupus erythematosus (SLE) adult donors.^[^ ^21^ ^]^ Using samples from healthy donors only, we derived differentially methylated cytosines (DMCs) between these monocyte subtypes, subsequently constructing a DNAm reference matrix over these DMCs (Figure S11a, Supporting Information, Methods). Adapting a hierarchical strategy^[^ ^29^ ^]^ that first estimates the total monocyte fraction, this reference matrix then allows us to quantify the relative proportions of classical and non‐classical monocytes in any blood sample (Methods). Validating this DNAm reference panel, we correctly predicted the 1202 CD14+CD16‐sorted monocyte samples from the MESA study^[^ ^30^ ^]^ to be predominantly classical (Figure S11b, Supporting Information), whilst also correctly assigning sorted classical and non‐classical monocytes from SLE donors into their respective subtypes (Figure S11c, Supporting Information). However, in the MESA study, a small number of samples also had a non‐negligible non‐classical fraction, and interestingly, this residual non‐classical fraction increased marginally with age (Figure S11d, Supporting Information), consistent with previous studies.^[^ ^31^, ^32^ ^]^ This increase in the non‐classical monocyte fraction with age was also seen in a large bulk DNAm dataset of PBMCs^[^ ^33^ ^]^ (Figure S11d, Supporting Information). The relative increase of non‐classical versus classical monocyte fraction with age was also recapitulated using two large scRNA‐Seq datasets, each with over 1 million peripheral blood mononuclear cells (PBMCs) from over 500 donors^[^ ^34^, ^35^ ^]^ (Figure S11e, Supporting Information). On the other hand, in a meta‐analysis over 14 whole‐blood DNAm datasets, we did not observe a consistent increase with age, except for some of the larger studies (Figure S11f, Supporting Information), highlighting that this age‐associated increase in the non‐classical monocyte fraction is a small effect and hence distinct of the age‐associated shift between young and adult monocytes. Moreover, when we applied UniLIFE to the previous DNAm dataset of sorted classical and non‐classical monocytes,^[^ ^21^ ^]^ we found that the relative estimates of adult and young monocytes was strongly skewed to the adult fraction in both classical and non‐classical subsets (Figure S11g, Supporting Information), a clear indication that our monocyte subtyping into young and adult phenotypes is distinct from the classical versus non‐classical one. Finally, a meta‐analysis over the same whole blood cohorts did not reveal consistent positive associations of the classical or non‐classical monocyte fractions with our CRP inflammaging score, with both fractions even correlating negatively with the IL‐6 score (Figure 3g). Thus, these data strongly support the view that our novel monocyte subtyping is orthogonal to the traditional classical versus non‐classical one, displaying stronger associations with inflammaging.

### 2.6 Age‐Related Monocyte Heterogeneity Correlates with Inflammaging in scRNA‐Seq and Metabolomic Data

**Figure 4:**
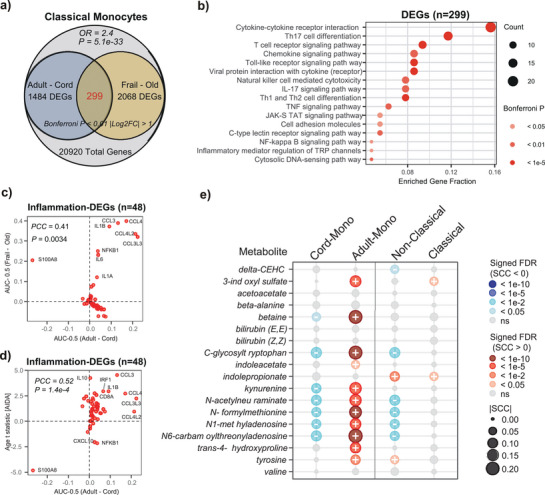
Age‐related monocyte subtyping correlates with inflammation in transcriptomic and metabolomic data. a) Venn diagram between genes differentially expressed between cord and adult blood classical monocytes, with genes differentially expressed in classical monocytes between old healthy and old frail individuals. Odds Ratio and P‐value of overlap from a one‐tailed Fisher test are given. b) Gene Set Enrichment Analysis (GSEA) of the 299 overlapping genes from a), displaying the enrichment of inflammation‐related KEGG pathways. A total of 48 inflammation‐related enriched genes were found. c) Scatterplot of AUC‐0.5 (derived from Wilcoxon rank sum test) for these 48 inflammation‐related genes between the adult versus cord blood classical monocytes (x‐axis) and between the frail and healthy old individuals (y‐axis). A Pearson correlation coefficient and P‐value are given. d) As c) but with the y‐axis now labeling the age‐associated t‐statistic as derived from the classical monocytes (n>200000) of the large AIDA scRNA‐Seq dataset. These statistics were derived from a linear mixed model including age and sex as fixed effects and donorID as a random effect. e) Balloon plot of Spearman Rank Correlation Coefficients (SCC) between inflammatory metabolites positively associated with IL‐6 levels with monocyte subtype fractions as estimated in the MGBB‐ABC cohort (n=1653). Cord and adult monocyte fractions were estimated using the 19 cell‐type UniLIFE panel, whereas the classical and non‐classical monocyte fractions were estimated using a 13 cell‐type panel.

To further demonstrate that subtyping monocytes by young and adult phenotypes is informative of inflammation, we turned to a single‐cell RNA‐sequencing (scRNA‐Seq) dataset profiling PBMCs from cord‐blood, adult blood from young and mid‐aged individuals, as well as adult blood from old healthy and age‐matched old frail individuals (Methods).^[^ ^36^ ^]^ We only considered the predominant classical monocyte population in order to demonstrate that the age‐related heterogeneity within this population is of biological relevance. First, we observed a significant overlap of genes differentially expressed between cord and adult classical monocytes (excluding old frail individuals) with genes differentially expressed between old healthy and old frail individuals (**Figure** **4**a). Second, the 299 overlapping genes were strongly enriched for inflammation‐related KEGG pathways (Figure 4b). Third, the subset of 48 inflammation‐related genes was consistently and predominantly overexpressed with both age and frailty (Figure 4c), thus demonstrating that age‐related heterogeneity within classical monocytes is informative of biological age. To demonstrate robustness, the same set of 48 genes was also predominantly positively correlated with age in the classical monocytes of the large AIDA scRNA‐Seq dataset (Figure 4d) which profiled over 200 000 classical monocytes in East Asians.^[^ ^35^ ^]^ Thus, scRNA‐Seq data support the view that age‐related heterogeneity within classical monocytes exists and that it is linked to inflammation and biological aging.

To more directly link our monocyte subtyping derived from DNAm data to inflammatory pathways, we next estimated the young/cord and adult monocyte fractions in an EPIC DNAm dataset from the Massachusetts General Brigham Aging Biobank Cohort (MGBB‐ABC) for which 1653 participants had matched metabolomics data (Methods).^[^ ^37^ ^]^ From a previous study, we obtained a list of metabolites that have been positively associated with IL‐6 levels,^[^ ^38^ ^]^ and asked if any of our estimated monocyte subtype fractions correlated with these same metabolites. Remarkably, these metabolites correlated consistently and positively with the adult monocyte fraction, whilst correlating negatively with the young (cord) monocyte fraction (Figure 4e, Tables S2 and S3, Supporting Information). In contrast, the classical monocyte fraction did not display significant correlations, with the non‐classical fraction only correlating negatively with a smaller number of metabolites (Figure 4e, Table S4, Supporting Information). Overall, these data confirm that our monocyte subtyping is biologically relevant, and further demonstrate how monocyte stratification into classical and non‐classical subtypes is far less informative of inflammaging compared to subtyping them by young and adult phenotypes.

### 2.7 Shifts Within the Innate Immune System Associate with Health Outcomes

**Figure 5:**
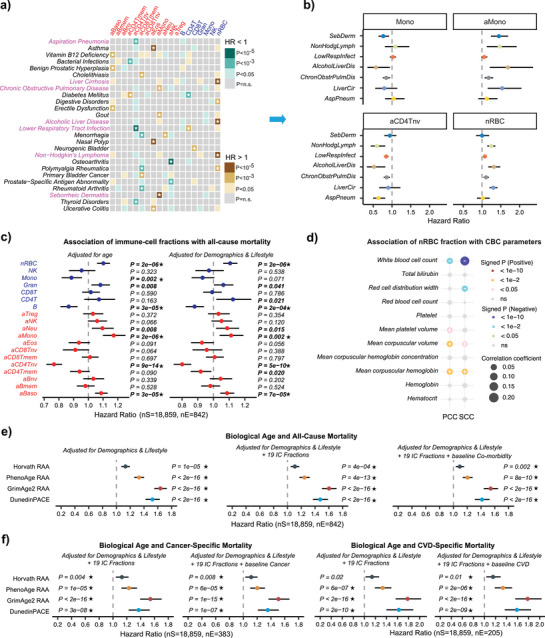
Associations of immune cell fractions with disease incidence and mortality in Generation Scotland. a) Heatmap of Cox‐regression Hazard Ratios (HR) and associated P‐values, between selected medical conditions and 19 immune cell‐type fractions in the Generation Scotland (GenS) cohort. b) Further subselection of medical conditions and immune cell‐fractions, displaying the precise HR values and their 95% confidence intervals. c) Forest plots of Cox‐regression HRs, 95% confidence intervals, and *P*‐values for the 19 immune cell‐type fractions in association with all‐cause mortality in GenS, with the left panel adjusted only for chronological age, and the right panel adjusted for age, other demographic and lifestyle risk factors. The number of samples (nS) and death events (nE) are given below the *x*‐axis. d) Balloon correlation plot displaying the correlations of the nRBC fraction with clinical complete blood cell (CBC) count measurements in the MGBB cohort. Correlation coefficients are indicated for both Pearson (PCC) and Spearman (SCC). e) Forest plots depicting the association of Horvath, PhenoAge, and GrimAge2 clock extrinsic age acceleration (EAA) and DunedinPACE with all‐cause mortality in the GenS cohort, using a 10‐year follow‐up window. The x‐axis labels the Hazard Ratio (HR), and 95% confidence interval is given for each estimate. The left panel is after adjustment for all demographic and lifestyle risk factors, the middle panel is when also adjusting for the 19 immune‐cell fractions, right panel is when additionally adjusting for baseline co‐morbidity (cancer, CVD, T2D, COPD, depression). The number of samples (nS) and death events (nE) are given below the *x*‐axis. f) As e) but for cancer and cardiovascular disease (CVD) specific mortality, adjusting for demographics, lifestyle, and 19 IC‐fractions (left panels) and additionally adjusting for baseline cancer/CVD status (right panels). *In all panels, associations are significant after Bonferroni adjustment for multiple testing.

Next, we assessed disease incidence associations of the 19 immune cell‐type fractions in the Generation Scotland (GenS) cohort (Methods, demographics in Table S5, Supporting Information), one of the largest available biobanks linked to extensive electronic health record data.^[^ ^39^, ^40^ ^]^ Using Cox‐proportional hazard regressions over a 10‐year follow‐up period after sample draw, we assessed correlations with 175 medical conditions, revealing a number of strong associations (Figure S12, Supporting Information). Confirming the reliability of the estimated immune‐cell fractions in GenS, we observed a very strong positive correlation between the adult eosinophil fraction with asthma (**Figure** **5**a), an association that is well established.^[^ ^41^, ^42^, ^43^ ^]^ The adult eosinophil fraction also correlated strongly with the presence of nasal polyps (Figure 5a), consistent with previous knowledge.^[^ ^44^, ^45^ ^]^ An increased adult naïve CD4T fraction was strongly associated with a reduced risk of respiratory conditions, including COPD and pneumonia, as well as non‐Hodgkin's Lymphoma (NHL), rheumatoid arthritis, and alcoholic liver disease (Figure 5a,b). A number of conditions, including dermatitis, COPD, and alcoholic liver disease, including liver cirrhosis, were associated with simultaneous increases and decreases of adult and young monocyte fractions, respectively, consistent with the ratio of adult to young monocytes being a marker of “inflammaging” (Figure 5b).

We also assessed associations with all‐cause mortality (Table S6, Supporting Information). A higher adult naïve CD4T fraction reduced the risk of all‐cause mortality independently of age, which remained significant after also adjusting for sex, BMI, alcohol, smoking, deprivation and education (Figure 5c), and baseline co‐morbidity (Figure S13a, Supporting Information), consistent with results obtained previously using the 12 adult immune‐cell DNAm reference panel on the Mass General Biobank (MGBB) cohort.^[^ ^18^ ^]^ Interestingly, the adult monocyte fraction was strongly associated with all‐cause mortality, whilst the young monocyte fraction displayed a negative association (Figure 5c, Figure S13a, Supporting Information), once again consistent with the relative proportions of these 2 cell‐types being a marker of “inflammaging,” which is known to be associated with the future risk of mortality.^[^ ^46^, ^47^ ^]^ In general, all these patterns were recapitulated when considering cancer or cardiovascular disease (CVD) specific mortality (Figure S13b, Supporting Information).

To validate these findings, we focused on another cohort, derived from the Mass General Brigham Biobank (MGBB) (Methods, demographics in Table S7, Supporting Information), that also has extensively annotated epidemiological and prospective health outcome information, including all‐cause mortality, type‐2 diabetes (T2D), cancer, chronic obstructive pulmonary disease (COPD), stroke, CVD and depression for a total of 3823 subjects. Among the 3823 subjects, there were a total of 430 death events. We used Cox‐proportional hazard regressions to evaluate associations between each of the 19 immune‐cell fractions with all‐cause mortality, adjusting for age, sex, race, and co‐morbidity at baseline, and then again by also adjusting for smoking, obesity, and alcohol consumption (Methods). Validating the findings in the GenS cohort, the naïve CD4+ T‐cell fraction decreased the risk of mortality, whereas the aMono fraction increased it (Figure S14, Supporting Information). Of note, using our previous lower resolution 12 immune‐cell type DNAm reference panel, we had not observed an association with mortality for the monocyte fraction.^[^ ^18^ ^]^ Thus, this further demonstrates the added value of our 19 IC‐type DNAm reference panel, as the relative proportion of adult to young monocytes not only correlates with inflammaging but also with all‐cause mortality, independently of competing covariates that include age, sex, BMI, alcohol, and smoking exposure.

### 2.8 A Non‐Negligible nRBC Fraction Increases with Age and Correlates with All‐Cause Mortality

Besides predicting non‐zero young innate immune cell fractions, our DNAm reference panel also predicted very small but non‐negligible nRBC fractions, which increased with age (Figure S3, Supporting Information), and which were also higher in men compared to women (Figure S4, Supporting Information). Whilst the nRBC fraction in the adult peripheral blood of a healthy individual is less than 1%, this fraction could be higher for older, unhealthy adults or adults with underlying medical conditions.^[^ ^48^ ^]^ Thus, it is plausible that our nRBC‐fraction is capturing low levels of circulating nRBCs or some other erythrocyte‐lineage cell. To exclude the possibility that our nRBC fraction may be measuring a missing cell‐type in our UniLIFE panel, we first used a recent large scRNA‐Seq study of 1.2 million PBMCs^[^ ^34^ ^]^ to identify hematopoietic progenitor cells (HPCs), plasmablasts, and dendritic cells as candidates, since all of these are more abundant than nRBCs in blood. Thus, we assembled a collection of independent Illumina DNAm datasets profiling sorted CD34+ hematopoietic stem and progenitor cells, as well as dendritic cells (Methods). Applying UniLIFE to each of these datasets revealed that HSCs and various forms of HPCs can be decomposed into cell‐types representative of myeloid, lymphoid, and erythrocyte lineages, with proportions mirroring the cell‐type's differentiation potential (Figure S15, Supporting Information). Most importantly, the estimated nRBC fraction was always relatively small at ≈10–15%, a clear indication that our nRBC profile is not representative of HSCs or other HPCs, the only exception being megakaryocyte‐erythrocyte progenitors (MEPs), i.e., the progenitor that gives rise to nRBCs, for which the estimated nRBC fraction was ≈30% (Figure S15, Supporting Information). Also, as expected, immature and mature dendritic cells were predicted to be most similar to monocytes (Figure S15, Supporting Information). Moreover, using the plasmablast fraction estimator (Methods),^[^ ^6^, ^13^ ^]^ its’ strongest association was with the neutrophil fraction, not with nRBCs (Figure S16, Supporting Information). Detailed inspection of the DNAm values of nRBC markers in our UniLIFE panel across sorted HPCs clearly revealed relatively big differences in DNAm between nRBCs and HPCs, again with the sole exception of MEPs (Figure S17, Supporting Information). Thus, based on all of this, we conclude that our estimated nRBC fraction is not capturing plasmablasts, dendritic cells, or HSC/HPCs, although we can't exclude that it is capturing a small component of circulating MEPs.

To further demonstrate that our algorithm is sensitive enough to detect very low nRBC fractions in adult blood, we performed an in‐silico spike‐in experiment, generating in‐silico mixtures of sorted blood cell‐types, including independently sorted nRBC samples (Methods). The fraction of nRBC cells in the in‐silico mixtures was varied from 0% to 10% in bins of 0.1%, 0.5%, 1% and 2% (Methods). Although our algorithm consistently overestimated nRBC fractions when concentrations were under 1% (Figure S18, Supporting Information), it was able to infer the true inter‐sample variation in nRBC fraction, even within a small dynamic range (say when nRBC fractions varied between 0.5% to 1%), achieving R‐values of 0.5 (Linear regression P<2e‐16, Figure S17, Supporting Information). Thus, despite the overestimation, the true variation in nRBC fraction between samples was correctly recapitulated (Figure S18, Supporting Information). This suggests that whilst our non‐zero nRBC fractions obtained in real adult blood cohorts are overestimates, the inferred variation between samples may be biological. Supporting this, the nRBC fraction displayed a strong correlation with inflammaging (Figure 3d) and with the risk of liver cirrhosis, COPD, and non‐Hodgkin's lymphoma (NHL) (Figure 5a,b). Notably, the nRBC fraction also displayed the second most significant association (Cox regression, P = 2e‐6) with all‐cause mortality, with higher estimates in this fraction increasing the risk of mortality independently of all major risk factors and baseline co‐morbidity (Figure 5c, Figure S13a, Supporting Information). Of note, this association with mortality was also independent of the years of follow‐up considered (Figure S19, Supporting Information).

### 2.9 nRBC Fraction Associates with Measures of Dysfunctional Erythropoiesis

Although it is well known that the circulating nRBC fraction is significantly increased in terminally ill dying patients,^[^ ^48^ ^]^ the observed correlation with the future risk of mortality, even up to 10 years before demise, suggests that this fraction may be capturing a chronic effect of increased dysfunctional erythropoiesis with age, that is further aggravated in individuals at higher risk of death. Indeed, dysfunctional erythropoiesis has been associated with all‐cause mortality.^[^ ^49^, ^50^ ^]^ In order to test if our nRBC fraction is capturing aspects of dysfunctional erythropoiesis, we correlated the nRBC fraction in the MGBB cohort to measures of erythropoiesis derived from a complete blood cell (CBC) count in a subset of ≈1600 individuals with matched DNAm and CBC data (Methods, Table S8, Supporting Information). Interestingly, we observed significant positive correlations with the mean corpuscular volume (MCV) (i.e., average size of RBCs) and mean corpuscular hemoglobin (Figure 5d), consistent with MCV's association with all‐cause mortality.^[^ ^49^, ^50^ ^]^ The nRBC fraction also correlated negatively with the white‐blood‐cell count (Figure 5d). Consistent with the nRBC fraction capturing nucleated DNA, it did not correlate with the enucleated red blood cell count, nor with hemoglobin or hematocrit (Figure 5d). Thus, overall, these data support the view that the measured nRBC fraction is a marker of dysfunctional erythrocyte function.

### 2.10 Epigenetic Clock Associations with All‐Cause Mortality are Independent of 19 IC Fractions

The strong association of the innate and nRBC cell subtypes with all‐cause mortality raises the question as to whether the reported associations of epigenetic clock acceleration measures with mortality are driven by these variations. To investigate this in the GenS cohort, we first verified that age acceleration, as measured by Horvath, PhenoAge, GrimAge2, and DunedinPACE clocks, was significantly associated with the 19 IC type fractions, with a pattern highly consistent with the one derived earlier from our meta‐analysis (Figure S20, Supporting Information). Age‐acceleration of these clocks, adjusted for all main demographic and lifestyle risk factors, was associated with all‐cause mortality, and these associations remained significant after additionally adjusting for the 19 immune‐cell fractions and baseline co‐morbidity (Figure 5e, Table S9, Supporting Information). Associations were marginally stronger for cardiovascular‐disease specific mortality compared to cancer‐specific mortality (Figure 5f). Overall, this suggests that, although immune‐cell type composition variation contributes to clock acceleration measures, health outcomes, and mortality risk, clock age‐acceleration measures contain a cell‐type composition independent component that remains informative of mortality risk.

## 3 Discussion

This work advances our understanding of epigenetic clocks by demonstrating how shifts within certain innate immune cell types correlate with epigenetic age‐acceleration estimates. This extends similar findings obtained for T‐cell lymphocytes to the innate immune system. In the case of adult CD4+ and CD8+ T‐cells, the naïve subsets effectively represent a “young” antigen‐inexperienced phenotype, as indeed they clustered more closely with their cord‐blood counterparts than with their adult memory ones. In the case of innate immune cell‐types, the cord‐blood subtypes can be viewed as representing a “young” phenotype, and as shown here, it is the relative fractions between the young and old phenotypes that correlate with epigenetic clock acceleration estimates, as well as being of biological and clinical significance. For instance, we have seen how the ratio of old to young monocyte subtypes in adult blood correlates with DNAm‐based surrogates of hsCRP (InflScore) and IL6 levels, and thus with inflammaging, a result which we validated in the MGBB‐ABC cohort using matched metabolomic data. Importantly, these monocyte subsets displayed much stronger consistent associations with inflammaging than classical/non‐classical monocyte fractions, a result seen in both the DNAm as well as metabolomic data. That our monocyte subtyping is orthogonal to the classical versus non‐classical dichotomy, whilst still capturing age‐related heterogeneity and features of inflammaging was further validated using transcriptomic data. Moreover, the adult monocyte fraction displayed a strong positive correlation with all‐cause mortality in both GenS and MGBB cohorts, even after adjusting for all major risk factors, including age, sex, smoking, body mass index, alcohol consumed, socioeconomic status, and baseline co‐morbidities. Given the potential of cord‐blood therapy to treat hematological, metabolic, and neurological conditions,^[^ ^51^ ^]^ our finding that a younger monocyte profile is protective of all‐cause mortality is of profound significance.

By estimating fractions for 19 immune cell‐types in 15 adult blood cohorts and correlating them to the InflScore, we have also characterized CRP‐related inflammaging in terms of specific changes in these 19 immune‐cell fractions. Besides the increased adult and decreased young monocyte fractions, inflammaging was also associated with an increased memory B‐cell fraction (consistent with lifelong antigen exposure), an increased memory and decreased naïve CD4+ T‐cell fractions (immune‐senescence) and an increased adult and decreased young NK‐cell fractions, which would be consistent with a more mature NK‐form being linked to inflammasome activation.^[^ ^52^ ^]^

We also found that the nRBC fraction correlated with inflammaging, with specific diseases like liver cirrhosis, as well as with all‐cause mortality, even after adjusting for all major risk factors. This is an intriguing and surprising finding since the nRBC fraction in circulation is thought to be negligible. Although our in‐silico spike‐in simulation confirmed that our estimated nRBC fractions are likely to be inflated, it nevertheless also showed that the relative variation in this fraction, although small, could be of biological significance, in line with our findings. Technically, this is plausible because erythrocytes display a very distinct DNAm profile to white blood cells, and because the relatively large number (i.e., n∼100) of nRBC‐specific markers in our UniLIFE panel can yield the power to detect subtle variations in this small fraction. It is thus conceivable that inflammaging may also be associated with a form of dysfunctional erythropoiesis in the bone marrow, which results in a non‐negligible fraction of nRBCs or erythroblasts/MEPs in circulation. Supporting this interpretation, in the MGBB cohort we found a strong correlation of the nRBC fraction with measures of dysfunctional erythropoiesis, including notably the mean cell volume (MCV), which is consistent with recent studies implicating aspects of erythrocyte biology in determining the risk of mortality.^[^ ^49^, ^50^, ^53^ ^]^ First of all, youthful erythrocyte function and metabolism have been shown to promote longevity by counteracting tissue hypoxia, inflammation, and oxidative stress.^[^ ^53^ ^]^ Second, the autophagy‐associated metabolite spermidine, which in blood tissue is mostly produced by erythrocytes, has a strong heritable component, with centenarians displaying higher than expected levels.^[^ ^49^ ^]^ Third, this same study also showed how spermidine levels strongly anti‐correlate with erythrocyte MCV, which itself is a risk marker for all‐cause mortality.^[^ ^49^, ^50^ ^]^ Moreover, erythrocyte MCV is particularly associated with liver cancer mortality,^[^ ^50^ ^]^ which is especially intriguing given that our nRBC (or erythroblast) fraction also correlated with liver cancer risk factors, including liver cirrhosis and alcoholic liver disease. Indeed, the pattern of correlations of the nRBC fraction with CBC variables, including an absence of a positive correlation with red cell distribution width, is suggestive of non‐megaloblastic macrocytic anemia, with one cause of this macrocytic anemia being alcohol misuse and liver cirrhosis (and another being myelodysplastic syndromes). Of note, this may explain why we detected the association of nRBC fraction with all‐cause mortality in the GenS cohort but not in MGBB, since the GenS cohort contained a much higher frequency of heavy+former drinkers (Tables S2 and S3, Supporting Information). Alternatively, the larger number of samples and events (n = 18859 samples and 842 death events) in GenS compared to the 3823 samples and 430 death events in MGBB, as well as the much longer follow‐up time in GenS (average 14 years) compared to MGBB (average 6 years), may underlie its increased power to detect this subtle association. Overall, it is thus highly plausible that a non‐negligible nRBC‐like fraction in adult blood, as estimated using our UniLIFE DNAm reference panel, is a proxy for dysfunctional erythrocyte function, which could manifest itself in various forms, including a larger MCV or an increased fraction of erythrocyte‐like cells.

Our work not only highlights how variations in the composition of innate and erythrocyte blood compartments correlate with inflammaging, health outcomes, and mortality, but also that these fractions correlate with age‐acceleration measures from epigenetic clocks. Associations of our monocyte subsets were particularly well correlated with DunedinPACE, which is among the most consistent predictors of biological age,^[^ ^54^ ^]^ as also assessed in interventional anti‐aging studies.^[^ ^55^ ^]^ Although the clocks themselves are associated with health outcomes and mortality, it is interesting that their associations with mortality were also independent of variations in the 19 immune cell‐type fractions. Thus, this extends previous findings obtained at the resolution of 6‐12 immune cell‐types^[^ ^4^, ^6^ ^]^ to a much higher cellular resolution, further underscoring how variations in blood cell type composition are only one biologically and clinically relevant component of epigenetic clocks, and hence that other DNAm‐changes, unrelated to cell‐type composition, are contributing to the associations with health outcome and mortality.

There are of course, a number of potential caveats to this study. First of all, the inflated nRBC fractions could be driven by original contamination of the sorted nRBC samples used in our DNAm reference panel construction. In other words, the original sorted nRBC samples may only be ≈80% pure, being contaminated with leukocytes, which would thus cause inflated estimates. However, it is worth noting that when UniLIFE is applied to cord blood DNAm datasets, estimated nRBC fractions are in line with those obtained using flow cytometry.^[^ ^24^ ^]^ Consistent with our in‐silico spike‐in experiment, the inflation of the nRBC fraction becomes negligible as the true fraction of nRBCs in the sample increases to ≈10% or higher. This suggests that contamination of the original sorted nRBC samples is not substantial.

A second potential caveat is the inherent assumption in our analysis that we are not missing a distinctive immune cell‐type in our DNAm reference panel, whose fraction in blood is non‐negligible and which could therefore skew estimated fractions, or induce spurious associations for other immune cell‐types (owing to the fact that estimated fractions need to add to 1). For instance, the proportion of hematopoietic progenitor cells (HPCs), dendritic cells (DCs), or plasmablasts in circulation is higher than that of erythrocytes.^[^ ^34^ ^]^ However, using DNAm profiles of sorted HPCs and DCs, we could confidently discard DCs as causing a skewing or confounding of nRBC estimates, although the presence of HPCs could marginally have inflated nRBC fractions. On the other hand, not having DCs in our DNAm reference panel could potentially also drive some of the variation between the young and adult monocyte subtypes. Plasmablasts, being a precursor of activated B‐cells, could be captured by our naïve or cord‐blood B‐cell components, and are unlikely to be a confounding factor. A more likely scenario, supported by our analysis of sorted cells, is that the nRBC fraction is capturing megakaryocyte‐erythrocyte progenitors (MEP), a direct precursor of nRBCs.

A third caveat is that although our novel monocyte subtyping showed a much stronger association with inflammaging than the classical versus non‐classical stratification, the latter is also a suboptimal benchmark, since monocytes in blood also encompass intermediate classical and immature phenotypes. Moreover, it remains to be seen if the cord‐blood monocyte represents an immature or reparative phenotype. Addressing these challenges will require unbiased multi‐modal single‐cell transcriptomic, proteomic, and DNAm approaches that can fully characterize the underlying heterogeneity,^[^ ^56^, ^57^, ^58^, ^59^ ^]^ allowing more complete and specific blood DNAm reference panels to be built.

In conclusion, this work highlights the critical importance of elucidating the heterogeneity of the innate immune system alongside the adaptive one to better interpret the associations of epigenetic clocks with health outcomes. Moreover, we have shown the added value of our UniLIFE DNAm reference panel in estimating young and adult innate immune cell‐type proportions that correlate with inflammaging, and, in the case of monocytes, also with all‐cause mortality. Furthermore, UniLIFE predicts non‐negligible fractions of nRBC‐like cells in blood that increase with age and inflammaging, and that also correlate with all‐cause mortality, pointing toward aberrant erythropoiesis or erythrocyte function as an important biomarker of health and lifespan. The UniLIFE DNAm reference matrix is part of our EpiDISH R‐package, which is freely available from https://www.bioconductor.org/packages/devel/bioc/html/EpiDISH.html or https://www.github.com/sjczheng/EpiDISH

## 4 Experimental Section

### Human Adult Whole Blood DNA Methylation Datasets of 15 Cohorts

All the cohorts used here have previously been normalized and analyzed by us,^[^ ^18^ ^]^ including: 1) *LiuMS (GSE106648)*: 279 HM450k peripheral blood (PB) samples, with age range from 16 to 66 (140 multiple sclerosis patients + 139 controls)^[^ ^60^ ^]^; 2) *Song (GSE169156)*, 2052 normal EPIC PB samples, with age range from 18 to 66^[^ ^33^ ^]^; 3) *HPT‑EPIC & HPT‑450k (GSE210255 & GSE210254)*: 1394 normal PB samples (EPIC set, age range from 21 to 87) and 418 normal PB samples (450k set, age range from 34 to 91)^[^ ^61^ ^]^; 4) *Barturen (GSE179325)*: 574 normal EPIC PB samples, with age range from 19 to 103^[^ ^62^ ^]^; (5) *Airwave (GSE147740)*: 1129 normal EPIC PB samples, with age range from 25 to 60^[^ ^63^ ^]^; 6) *VACS (GSE117860)*: 529 HM450k samples from whole blood in HIV‐positive men, with age range from 25 to 75^[^ ^64^ ^]^; 7) *Ventham (GSE87648)*: HM450k PB samples from 204 newly‐diagnosed IBD cases and 178 controls, with age range from 17 to 79, and 2 samples have no age information^[^ ^65^ ^]^; 8) *Hannon‑1 and 2 (GSE80417 & GSE84727)*: 675 normal HM450k PB samples with age range from 18 to 90, and 847 normal HM450k PB samples with age range from 18 to 81^[^ ^66^, ^67^ ^]^; 9) *Zannas (GSE72680)*: 422 normal HM450k PB samples, with age range from 18 to 77^[^ ^68^ ^]^; 10) *Flanagan/FBS (GSE61151)*: 184 normal HM450k PB samples, with age range from 35 to 83^[^ ^69^ ^]^; 11) *Johansson (GSE87571)*: 732 normal HM450k PB samples, with age range from 14 to 94, and 3 samples have no age information^[^ ^70^ ^]^; 12) *TZH (OEP000260)*: 710 normal EPIC PB samples, with age range from 19 to 71^[^ ^71^ ^]^; 13) *HNM (GSE40279)*: 656 normal HM450k PB samples, with age range from 19 to 101.^[^ ^9^ ^]^ 14) *LiuRA* (*GSE42861*): HM450k PB samples from 354 Rheumatoid arthritis cases and 335 controls, with age range from 18 to 70.^[^ ^72^ ^]^

### Human Umbilical Cord Blood and nRBC DNA Methylation Datasets

*GSE149572*: An Illumina HM450k dataset of 3 human adult and 20 human cord blood samples,^[^ ^73^ ^]^ previously normalized and analyzed by Guo et al.^[^ ^24^ ^]^

*GSE82084*: An Illumina 450K dataset profiling T cells, monocytes, granulocytes, and nucleated red blood cells (nRBCs) isolated from cord blood of term and extreme preterm (<31 weeks gestational age) individuals.^[^ ^74^ ^]^ 36 samples in total: 5 each of T cells, monocytes, granulocytes, and nRBCs from term births; and 5 T cells, 4 monocytes, 4 nRBCs, and 3 granulocytes from preterm births.

### Estimation of Cell‑Type Fractions

In all cohorts, given the UniLIFE DNAm reference matrix for either the Illumina EPIC or 450k dataset, corresponding cell‐type fractions were estimated using the EpiDISH Bioconductor R‐package. Specifically, the *epidish* function was run with “RPC” as the method and *maxit* = 500. The abundance of plasmablasts was estimated using Horvath's Online DNAmAge Calculator, available at https://dnamage.clockfoundation.org.

### DNAm‐Based CRP‐Score (InflScore)

Wielsher et al. identified 1765 marker CpGs significantly associated with serum C‐Reactive protein levels at a Bonferroni threshold (P < 1e−7) in their multi‐ethnic meta‐analysis,^[^ ^25^ ^]^ having adjusted for the fractions of 6 immune cell types. To apply this signature to other DNAm datasets, each CpG was first z‐score normalized across samples in each cohort so that the mean is 0 and the standard deviation is 1. The sign of each CpG's effect size defines a vector that is then correlated (Pearson) to the z‐score normalized DNAm profiles of each sample to yield a sample‐specific inflammation score (InflScore).

### DNAm‐Based IL‐6 Score

Stevenson et al. developed a DNAm‐based predictor of serum interleukin‐6 (IL‐6) levels from a cohort of 875 older adults, using Elastic Net regression, where serum IL‐6 level was used as the dependent variable and the CpGs from the 450k array were used as the independent variables.^[^ ^26^ ^]^ However, they did not adjust for cell type fractions. This predictor comprises 35 CpGs and their regression coefficients.^[^ ^26^ ^]^ This predictor was applied across the blood DNAm datasets to obtain the IL‐6 DNAm‐based score of each sample.

### DNA Methylation Clocks

The dnaMethyAge R package was used to calculate each individual's Horvath age and PhenoAge.^[^ ^75^ ^]^ Then, these were regressed against chronological age to obtain residuals as measures of Relative Age Acceleration (RAA). DunedinPACE was estimated using the PACEProjector() function from the R package DunedinPACE.^[^ ^12^ ^]^ GrimAge2 was calculated following.^[^ ^5^ ^]^

### Meta‐Analyses

In each cohort, we standardized the fractions of each cell type to unit variance. Associations between the standardized cell type fractions and other phenotypes (e.g., age, sex, RAA, and InflScore) were then assessed using a multivariate linear regression. For specific cohorts where additional covariates were available, such as gender, disease, and smoking status, the multivariate regression models include these factors as covariates. The covariate information used for each cohort is described in detail in Luo et al.^[^ ^18^ ^]^ For each regression, the corresponding effect size, standard error, Student's *t*‐test, and *P*‐value were extracted. A fixed and random effect inverse variance meta‐analysis was then performed using the metagen function implemented in the meta R‐package (version 7.0‐0).^[^ ^76^ ^]^

### Monocyte DNAm Datasets and Construction of Classical Versus Nonclassical DNAm Reference

An EPIC dataset was downloaded from the GEO data repository (GEO accession GSE249641),^[^ ^21^ ^]^ profiling 137 samples representing sorted classical monocytes, intermediate monocytes, and non‐classical monocytes from 20 SLE patients at flare‐up and follow‐up visits, and 10 healthy donors. After downloading the IDAT files from GEO, they were preprocessed using the `preprocessRaw` function from the `minfi` package. Probes on sex chromosomes, SNP probes, cross‐reactive probes, and probes with a *p*‐value > 0.01 in any sample were removed, leaving 773697 probes. Type‐2 bias was corrected using BMIQ. Another DNAm dataset of sorted CD14+ monocytes from MESA‐study (n = 1202 samples)^[^ ^30^ ^]^ was previously normalized and analyzed in Luo et al.^[^ ^18^ ^]^ This dataset is available from GEO: GSE56046, and age ranges from 44 to 83.

Using Ferrete‐Bonastre's dataset,^[^ ^21^ ^]^ the monocyte subtype reference panel was constructed from 10 classical monocyte samples and 8 non‐classical monocyte samples from healthy donors. A performed differential DNAm analysis was then performed using limma to compare these two monocyte subtypes, identifying 4224 hypermethylated DMCs for classical monocytes (hypomethylation for non‐classical monocytes) with a threshold of FDR < 0.05. These DMCs were then ranked using the gap specificity score.^[^ ^24^ ^]^ By setting a threshold of 0.3, we identified 60 highly discriminative marker CpGs. The final DNAm reference matrix, defined over these 60 marker CpGs and the two monocyte subtypes, was built by taking the mean value over the samples of a given cell type.

For validation purposes, EpiDISH was directly applied using the robust partial correlations method (RPC, as implemented in EpiDISH, maxit = 500) with this two‐cell‐type DNAm reference panel to estimate the proportions of two monocyte subtypes for sorted classical and non‐classical monocytes from SLE patients. To obtain the monocyte subtype fractions in the sorted monocyte MESA study data, we also directly applied this 2‐cell‐type DNAm reference panel using EpiDISH. For all other whole blood DNAm datasets, the previously established 12 adult blood cell‐type reference panel + EpiDISH was used to first estimate the proportion of total monocytes. In the second step, the 2‐cell‐type monocyte reference panel was applied to estimate the classical versus non‐classical subfractions. The final fractions are then given by the product of the total monocyte fraction multiplied by the relative fraction of each monocyte subtype.

### scRNA‐Sequencing Datasets Used for Monocyte Analysis

We used the scRNA‐Seq dataset from Luo et al. (https://www.ncbi.nlm.nih.gov/geo/query/acc.cgi?acc=GSE157007), which contains mononuclear cells from umbilical cord blood (n = 3), peripheral blood mononuclear cells (PBMCs) from young/mid‐aged adults (n = 3; age, 30.7 ± 10.0 years), from healthy old (n = 6; age, 85.8 ± 11.1 years), and old frail (n = 5; age, 88.0 ± 5.8 years) individuals.^[^ ^36^ ^]^ Raw count data were processed following the standard Seurat v5 workflow.^[^ ^77^ ^]^ To identify differentially expressed genes (DEGs), the analysis was performed exclusively on classical monocytes, as annotated by the authors. The Wilcoxon rank‐sum test was applied for the following comparisons: 1) cord blood versus the adult group (excluding the old frail), and 2) old healthy versus old frail individuals. Genes were considered significantly differentially expressed if they met the criteria of a Bonferroni‐adjusted *P*‐value < 0.05 and an absolute log_2_(fold change) > 1. To identify biological functions robustly associated with both aging and frailty, the set of DEGs common to both comparisons (i.e., the intersection of DEGs from the “cord blood versus young/old” and “old versus frail”) was first identified. This core set of shared DEGs was then subjected to KEGG pathway enrichment analysis using the clusterProfiler R package.^[^ ^78^ ^]^ Pathways with a Bonferroni‐adjusted *P*‐value < 0.05 were considered significantly enriched. Following this, the investigation focused on a curated list of inflammation‐related pathways, which were selected based on their well‐documented roles in the relevant literature. The constituent genes of the identified pathways were analyzed in an independent cohort of 202124 classical monocytes from the “AIDA” dataset (https://cellxgene.cziscience.com/collections/ced320a1‐29f3‐47c1‐a735‐513c7084d508), which includes 595 individuals aged 19‐77.^[^ ^35^ ^]^ A linear mixed model was fitted for each gene using the lmer function (lme4 package) with age and sex included as fixed effects, and Subject_ID as a random effect to ensure each subject contributes equally to the model, thereby mitigating potential biases caused by the varying number of cells per individual.

To assess if the ratio of non‐classical and classical monocytes changes with age, the scRNA‐Seq dataset from Yazar et al.^[^ ^34^ ^]^ was used, which is larger than AIDA, comprising 1248980 peripheral blood mononuclear cells (PBMCs) obtained from 981 donors. The data, structured as a Seurat Object, was downloaded from https://cellxgene.cziscience.com/collections/dde06e0f‐ab3b‐46be‐96a2‐a8082383c4a. This dataset incorporates extensive metadata, including annotations for 29 distinct PBMC subtypes, corresponding sample identifiers, donor age, sex, and UMAP coordinates for each cell. The analysis concentrated on two specific monocyte subtypes: classical monocytes and non‐classical monocytes, with 36130 and 15743 cells, respectively. The donors with a total monocyte count below 30 were excluded. The relative fraction of each monocyte subtype per donor was subsequently computed, defined as the proportion of a given subtype relative to the donor's total monocyte count. To account for potential confounding variables, such as sex and batch (i.e., pool number), a linear model was employed to adjust the monocyte subtype fractions. This adjustment was achieved using the residuals from the model: adjusted fraction = residuals from lm(fraction∼Sex+pool_id). Subsequently, to ensure that the analysis was weighted according to the abundance of data per donor, a weighted linear regression was applied. Specifically, this regression model assigns greater weight to donors contributing a higher total number of monocytes, thereby enhancing the precision of our estimates: lm(adjusted_fraction∼age, weights=total number of monocytes). Both the linear adjustment and the weighted regression analysis were performed using the lm() function from the R‐package stats.

### Analysis of Generation Scotland Cohort Data

Generation Scotland (GenS) is one of the world's largest biobanks with access to blood DNAm samples linked to electronic health records.^[^ ^40^, ^79^ ^]^ GenS is a family‐based cohort of ≈24000 individuals from across Scotland. Volunteers were aged 17–99 years at the point of recruitment (2006–2011). Blood samples were taken at a baseline clinic visit, from which DNA methylation was successfully profiled for 18869 individuals via the Illumina EPIC array.^[^ ^80^ ^]^

A total of 308 disease outcomes, based on primary and secondary care codes as per Kuan et al.^[^ ^39^ ^]^ were defined for Generation Scotland participants. Data linkage to electronic health records spanned the period from January 1980 to April 2022. The time to the first event after blood draw for methylation was considered for each disease. Those with a prevalent event (ie, diagnosis before blood draw) were excluded from the analyses for that particular disease. Those who remained free of the outcome diagnosis up until April 2022 or who died without having received a diagnosis were censored. Death data were obtained via linkage to the National Records of Scotland. While consent to access primary care records was given by all participants, these were only accessible for ≈40% of the cohort due to consent constraints with the data holders (individual GP surgeries).

EpiDISH^[^ ^81^ ^]^ with the UniLIFE DNAm reference panel^[^ ^24^ ^]^ was applied to estimate fractions for the 19 immune cell‐types in the GenS EPIC data. An incidence disease analysis was then performed for all 19 immune‐cell fractions. The analysis focused on 175 of the 308 diseases for which at least 30 occurrences were observed. A pedigree kinship matrix was pre‐regressed from each of the immune cell fractions to account for relatedness in the cohort. Cox proportional hazards models were conducted for each immune‐cell proportion (scaled to mean zero and unit variance) and 10‐year onset for each of the 174 disease outcomes. Where the proportion of cases exceeded 90% for one sex, the regression was filtered to only consider individuals of that sex. Covariates included age, sex, body mass index (BMI, calculated as weight in kg divided by squared height in meters), smoking pack years (calculated by self‐reported packs per day multiplied by number of years as a smoker), alcohol units consumed over the last week, years of education (an ordinal variable with 11 categories: 1) 0 years, 2) 1–4 years, 3) 5–9 years, 4) 10–11 years, 5) 12–13 years, 6) 14–15 years, 7) 16–17 years, 8) 18–19 years, 9) 20–21 years, 10) 22–23 years, 11) 24 or more years), and an area‐based rank of socioeconomic deprivation (Scottish Index of Multiple Deprivation, SIMD). SIMD measures relative deprivation across 6976 data zones, calculated across seven domains: income, employment, education, health, access to services, crime, and housing. Alcohol, smoking pack years, and BMI were log transformed (a constant of 1 added to alcohol and smoking prior to transformation). Missing data were imputed via k‐nearest neighbors’ (k = 10). The maximal number of missing entries was 1734 for alcohol consumption. Covariates were standardized to a mean of zero and unit variance. Schoenfeld residuals were used to examine the proportional hazards assumption at the model level and also for the predictor (immune cell fraction) of interest. For all‐cause mortality, Cox proportional hazard regression models were run as above, but in two ways: adjusting only for age, and adjusting for all covariates as described above for the disease incidence analysis. Linear regression models were also run for immune cell fractions against the RAA of epigenetic clocks, whilst also adjusting for sex: lm(scale(clock) ∼ scale(WBC) + scale(age) + sex).

### Analysis of Mass General Biobank Data (MGBB)

A total of 4386 whole blood samples were retrieved from the MGB Biobank^[^ ^82^ ^]^ to study associations of the UniLIFE immune‐cell fractions with health outcomes. MGB derived DNA samples were processed by TruDiagnostic Inc. lab facility (Lexington, KY, USA) for DNAm profiling with EPICv1 arrays as described by us previously.^[^ ^18^ ^]^ We queried the demographic information (i.e., date of birth, sex, and ethnicity), health history (i.e., smoking status, alcohol consumption, and BMI), and clinical records (i.e., patient diagnosis) of the 4386 human donors from MGBB and The Research Patient Data Repository (RPDR) databases.^[^ ^83^ ^]^ The vital status (i.e., living/deceased) and date of death were also obtained from MGB Biobank. However, 147 subjects who were recorded as deceased had a missing date of death, and they were excluded from the survival analysis of all‐cause mortality. Other diseases, including type‐2 diabetes, chronic obstructive pulmonary disease (COPD), cardiovascular disease (CVD), cancer, and depression, were identified using relevant ICD‐9/10 diagnosis codes (referred to the supplement codebook). Missing data for smoking status, alcohol consumption, and BMI were imputed using longitudinal records of these variables. Specifically, for smoking status and alcohol consumption, the record closest to the sample collection date was used. For BMI, it is imputed that the missingness as the median value of all BMI records within 6 months around the collection date to balance off the measurement error and temporal variation. Despite imputation, 605 subjects still had missing BMI data and were excluded from the survival analysis when further adjusting for additional risk factors, including BMI. We estimated the hazard ratio of each immune cell type against all‐cause mortality using Cox proportional hazard regression models with *coxph* function in *survival* R package. The models were adjusted for age, sex, ethnicity, and baseline comorbidities (which included cancer, CVD, COPD, depression, and T2D), and separately again adjusting for age, sex, ethnicity, smoking status, alcohol consumption, BMI, and the same baseline comorbidities.

### In‐Silico Spike‐In Experiment for nRBC

To assess the sensitivity of the UniLIFE DNAm reference panel in detecting low fractions of nucleated red blood cells (nRBCs), an in‐silico spike‐in experiment was conducted using independent sorted Illumina DNAm data not employed in the reference panel's construction. For nRBCs, three samples with the highest purity (≈0.9, estimated by our UniLIFE reference) were selected from GSE82084.^[^ ^74^ ^]^ For the six immune cell types—neutrophils, monocytes, CD4 T‐cells, CD8 T‐cells, natural killer cells, and B‐cells—three samples per cell type with purity greater than 0.98 were selected from GSE224807, as estimated by the centDHSbloodDMC.m panel in EpiDISH. Both sorted datasets were previously normalized and analyzed by Guo et al.^[^ ^24^ ^]^ Realistic proportions for the six immune cell types were obtained from 1000 randomly selected samples in the HPT‐EPIC cohort, with cell type fractions estimated using the centDHSbloodDMC.m panel in EpiDISH. For nRBCs, 1000 random fractions were generated within each of nine bins: 0% (no nRBCs), 0–0.1%, 0.1–0.5%, 0.5–1%, 1–2%, 2–4%, 4–6%, 6–8%, and 8–10%. In each bin, 1000 proportions were constructed by combining the realistic proportions of immune cells from the HPT‐EPIC cohort with the random nRBC fractions. To ensure all cell type proportions summed to 1, the immune cell fractions were rescaled by multiplying them by (1‐nRBC fraction). Each mixture was generated by randomly selecting one sample per cell type and calculating the weighted average of their DNAm profiles based on the rescaled proportions. The UniLIFE reference panel was then applied to estimate nRBC fractions in these simulated mixtures, and the Pearson correlation coefficient between the true and estimated fractions was computed for each bin.

### Human DNA Methylation Datasets of HPCs and Dendritic Cells

*GSE40799*: 3 freshly isolated and 9 upon expansion CD34+ cell HM450k samples derived from cord blood.^[^ ^84^ ^]^ *GSE72867*: 69 sorted CD34+ cell HM450k samples from cord blood, including 15 untreated CD34+ cell profiles.^[^ ^85^ ^]^ *GSE59796*: 4 sorted monocytes and 8 dendritic cell HM450k samples from the blood of healthy donors.^[^ ^86^ ^]^ *GSE63409*: HM450k samples included 20 leukemia stem cells, 24 blast cells, and 30 normal hematopoietic stem and progenitor cells (6 different types from 5 normal bone marrows).^[^ ^87^ ^]^ The steps for processing these datasets include: i) Downloading IDAT files or pre‐processed data from GEO (IDAT files are not provided, but a beta matrix containing NAs is available). ii) Preprocessing IDAT files using the preprocessRaw function from the minfi package. iii) Removing probes on sex chromosomes, SNP probes, cross‐reactive probes, and probes with a *p*‐value > 0.01 in any sample. 4). Using BMIQ to adjust Type‐2 probe bias.

### Processing of Mass General Biobank (MGBB) Samples

Biobanked whole blood samples were stored at −80 °C until shipment to TruDiagnostic Inc. (Lexington, KY) for DNA extraction and array‐based methylation profiling. Genomic DNA (500 ng per sample) was extracted and bisulfite‐converted using the EZ DNA Methylation Kit (Zymo Research, Irvine, CA), following the manufacturer's protocol. Bisulfite‐converted DNA was randomized across chip positions on the Infinium HumanMethylationEPIC BeadChip array (Illumina). Laboratory preprocessing steps included DNA denaturation and amplification, hybridization to array probes, single‐base extension and staining, followed by washing and scanning using the Illumina iScan SQ system to produce raw fluorescence intensity data.

DNA methylation was profiled using the Illumina HumanMethylationEPIC array. A total of 4832 samples were processed as raw.idat files using the minfi and ENmix packages in R (version 4.2.2). Initial quality assessment was conducted using the qcReport() function in minfi, which generated summary plots and detection p‐value distributions per sample. Probes and samples were filtered based on detection *p*‐value thresholds: probes with detection p > 0.01 in more than 1% of samples were excluded, and samples with mean detection p > 0.05 were removed from further analysis, retaining 4756 samples. SNP‐associated probes (CpG or single base extension overlap) and loci known to cross‐hybridize were also removed using dropLociWithSnps() to reduce technical artifacts. Raw intensity data were converted to beta values via preprocessRaw() followed by dye‐bias and background adjustment using ratioConvert() and mapToGenome() within minfi. Probes with more than 10% missing beta values were removed, resulting in 822474 retained CpG sites. To impute the remaining missing values while managing memory overhead, we implemented a chunk‐wise k‐nearest neighbors (KNN) imputation strategy. The filtered matrix was split into sequential blocks of 10000 CpGs. Each block was imputed using impute.knn() (k = 5), and all chunks were recombined into a complete, imputed beta matrix. This approach preserved sample integrity while minimizing computational burden. To further correct for dye bias in Type II probes, we applied the Reference Channel Probe (RCP) normalization method. A custom implementation of the betaRCP() function was used, applying M‐value linear adjustment to harmonize the quantile distributions of Type I and Type II probes within proximity on the genome. RCP‐adjusted beta values were used for all downstream modeling. The RCP‐normalized, KNN‐imputed beta matrix served as input for reference‐based immune cell deconvolution using the 19 cell‐type UniLIFE reference using the EpiDISH() function, and using the RPC method and max iterations of 500.

### Correlation Analysis of Immune‐Cell Fractions to Clinical Biomarkers in MGBB

The Phenotype Discovery Center (PDC) of MGBB integrates various data sources, including Research Patient Data Registry (RPDR), health information surveys, and genotype results, into the Biobank Portal. This portal combines specimen data with EMR data, creating a comprehensive SQL Server database with a user‐friendly web‐based application.^[^ ^82^ ^]^ Available data for 11 CBC biomarkers were extracted from clinical tests performed on blood samples collected at the same visit as those used for DNAm profiling. Sample sizes for each biomarker are: Total bilirubin (N = 1114), Mean platelet volume (N = 1194), Platelet count (N = 1660), Red blood cell count (N = 1660), Mean corpuscular hemoglobin concentration (N = 1660), White blood cell count (N = 1660), Mean corpuscular volume (MCV, N = 1660), Mean corpuscular hemoglobin (N = 1660), Red cell distribution width (N = 1657), Hemoglobin (N = 1660), Hematocrit (N = 1660). To reduce skewness and stabilize variance, total bilirubin, red cell distribution width, and hemoglobin were natural‐log‐transformed before analysis. We calculated the Pearson and Spearman correlation coefficients between each hematologic biomarker and the nRBC fraction derived from the UniLIFE DNAm reference panel.

### Correlation Analysis of IC‐Fractions to Metabolites and Proteomics in MGBB

All the multi‐omics data were derived from a previously characterized subset of 1653 participants in the Massachusetts General Brigham Aging Biobank Cohort (MGB‐ABC), as described in detail elsewhere.^[^ ^37^ ^]^ DNA methylation profiling of this subset was performed using the Illumina EPIC array and processed as described previously.^[^ ^37^ ^]^ Briefly, raw IDATs were processed using the minfi package^[^ ^88^ ^]^ with functional normalization (Funnorm),^[^ ^89^ ^]^ followed by dye‐bias correction via the RCP method from the ENmix package.^[^ ^90^ ^]^ Sample‐and probe‐level quality control was performed using qcfilter() and detection p‐value thresholds (p < 0.05) as in Foox et al.^[^ ^91^ ^]^ Untargeted plasma metabolomics was performed by Metabolon Inc. using established LC‐MS protocols and rigorous quality control, as previously detailed.^[^ ^37^ ^]^ Briefly, four complementary chromatographic methods were used to capture a broad range of metabolite classes, with final feature selection and scaling procedures as described.^[^ ^37^, ^92^ ^]^ Duplicate samples were averaged, and outlier samples whose metabolite values were > 3 standard deviations away from the mean were removed. Proteomic data were acquired using the Seer Proteograph Product Suite and quantified via LC‐MS with data‐independent acquisition (DIA), as previously described.^[^ ^37^, ^93^ ^]^ Protein intensities were normalized using established workflows, including residualization on age, sex, and genotype‐derived principal components to adjust for covariates and technical artifacts.

### General Statistical Analysis

All statistical analyses were conducted using R software (version 4.4.0). Specific analyses relied on Bioconductor packages including limma and EpiDISH, as well as CRAN packages such as meta, survival, and lme4. Unless otherwise specified (e.g., one‐tailed Fisher's exact test), all statistical tests were two‐sided. Associations between variables were assessed using multivariate linear regression (lm) or linear mixed‐effects models (lmer for single‐cell data), while correlations were evaluated using Pearson and Spearman coefficients. Survival and disease incidence were analyzed with Cox proportional hazards regression models (coxph), with the proportional hazards assumption verified using Schoenfeld residuals. Differential analyses were performed using the Wilcoxon rank‐sum test or empirical Bayes moderated *t*‐tests (limma). To synthesize findings across cohorts, we employed random‐effects meta‐analysis (meta package). For analyses involving multiple comparisons, *P*‐values were adjusted using the Bonferroni method to control the family‐wise error rate. Significance was declared at P < 0.05 after Bonferroni adjustment. Specific sample sizes (n), the statistical tests used, and definitions for all significance symbols are provided in the relevant text or Figure legends.

### Ethics Approval and Consent to Participate

All components of Generation Scotland received ethical approval from the NHS Tayside Committee on Medical Research Ethics (REC Reference Number: 05/S1401/89). All participants provided broad and enduring written informed consent for biomedical research. Generation Scotland has also been granted Research Tissue Bank status by the East of Scotland Research Ethics Service (REC Reference Number: 15/0040/ES), providing generic ethical approval for a wide range of uses within medical research. All participants of MGBB cohort provided consent to use the collected data for this project. This study was performed in accordance with the Helsinki declaration.

## Author's Contribution

X.G., J.A.R., A.A., and K.S. contributed equally to this work. X.G. performed the statistical and bioinformatic analyses, with assistance of J.A.R., A.A., K.S., Z.D., V.D., Q.C., R.E.M., and A.E.T., B.T.H. and C.A.M.C. if contributed feedback. E.B. contributed data and pointers. R.S. contributed the DNAm MGBB data. J.L.S. contributed the epidemiological data from MGBB. S.H. contributed feedback and the GrimAge2 code. R.E.M. contributed the data from the GenS cohort. A.E.T. conceived the study and wrote the article with contributions from X.G., R.E.M., J.A.R., A.A. and K.S.

## Conflict of Interest

AET is a consultant for TruDiagnostics Inc. REM is an advisor to the Epigenetic Clock Development Foundation and Optima Partners Ltd. SH is a founder and paid consultant of the non‐profit Epigenetic Clock Development Foundation that licenses these patents. SH is a Principal Investigator at the Altos Labs, Cambridge Institute of Science.

## Supporting information



Supporting Information

Supporting Information

## Data Availability

The following DNAm datasets analyzed here are publicly available from the NCBI GEO website https://www.ncbi.nlm.nih.gov/geo/ under the accession numbers GSE40279 (HNM), GSE106648 (LiuMS), GSE169156 (Song), GSE210255 (HPT‐EPIC), GSE210254 (HPT‐450k), GSE179325 (Barturen), GSE147740 (Airway), GSE117860 (VACS), GSE87648 (Ventham), GSE84727 (Hannon2), GSE80417 (Hannon1), GSE72680 (Zannas), GSE61151 (Flanagan/FBS), GSE87571 (Johansson), GSE42861 (LiuRA), GSE249641 (Ferrete‐Bonastre), GSE56046 (Reynolds), GSE82084 (deGoede), GSE224807 (Bell), GSE40799 (CD34+), GSE72867 (CD34+), GSE59796 (DCs), GSE63409 (HPCs) and GSE149572 (Cord blood). The Illumina EPIC DNAm data for the TZH cohort can be viewed at NODE under accession number OEP000260, or directly at (https://www.biosino.org/node/project/detail/OEP000260), and accessed by submitting a request for data‐access. The scRNA‐Seq data from Yazar et al is publicly available from https://cellxgene.cziscience.com/collections/dde06e0f‐ab3b‐46be‐96a2‐a8082383c4a1. Instructions for accessing Generation Scotland data can be found here: https://www.ed.ac.uk/generation‐scotland/for‐researchers/access; the ‘GS Access Request Form’ can be downloaded from this site. Completed request forms must be sent to access@generationscotland.org to be approved by the Generation Scotland Access Committee. The DNA methylation data of MGBB samples are not publicly available due to participant privacy and institutional policies. Researchers interested in accessing the data may contact varun.dwaraka@trudiagnostic.com. Data will be made available upon reasonable request and completion of a data usage agreement (DUA) approved by TruDiagnostic Inc.
