# Supplementary material for: Variations in Innate Immune Cell Subtypes Correlate with Epigenetic Clocks, Inflammaging and Health Outcomes
Source: Adv Sci (Weinh). 2025 Aug 27;12(43):e05922. doi: 10.1002/advs.202505922 (PMC12631840; doi:10.1002/advs.202505922)
Supplement: Supplementary file 1 — Supporting Information [file ADVS-12-e05922-s001.pdf]

Supplementary Figures

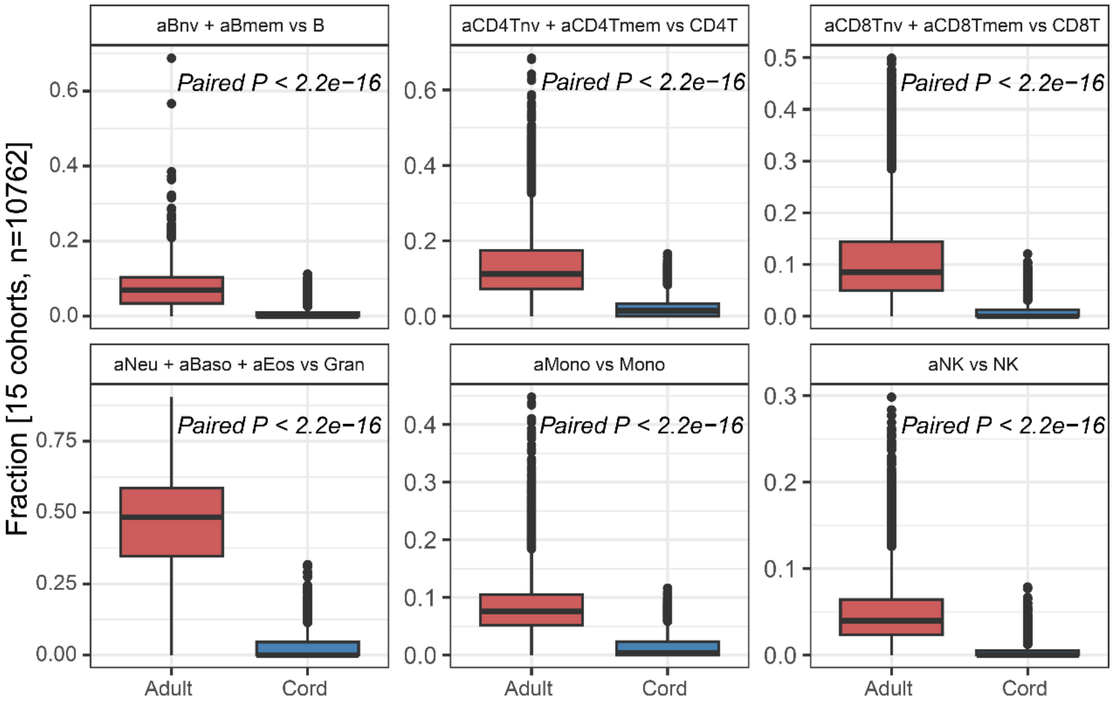

SI fig.S1: UniLIFE fractions of adult immune cell-types are higher than corresponding cord-blood ones across 15 whole blood cohorts. For six immune cell-types, we compare the UniLIFE estimated fractions of the adult subtype (red boxplots) against the cord-blood subtype (blue) across 15 adult whole blood cohorts, encompassing 10,762 samples, i.e. the number of samples in each boxplot is 10,762. The P-value is from a paired one-tailed Wilcoxon rank sum test, since for every sample we can compare the adult and cord-blood fraction. Hence this is adjusted for batch (cohort). In the case of B-cells, we are comparing the sum of adult naïve and memory B-cells (aBnv+aBmem) to cord-blood B-cells (B). Similarly, for CD4+ and CD8+ T-cells. For monocytes (Mono) and natural killer (NK) cells we compared adult to cord-blood. For granulocytes, we compare the sum of adult neutrophil, basophil and eosinophil fractions (aNeu+aBaso+aEso) to cord-blood granulocytes (Gran).

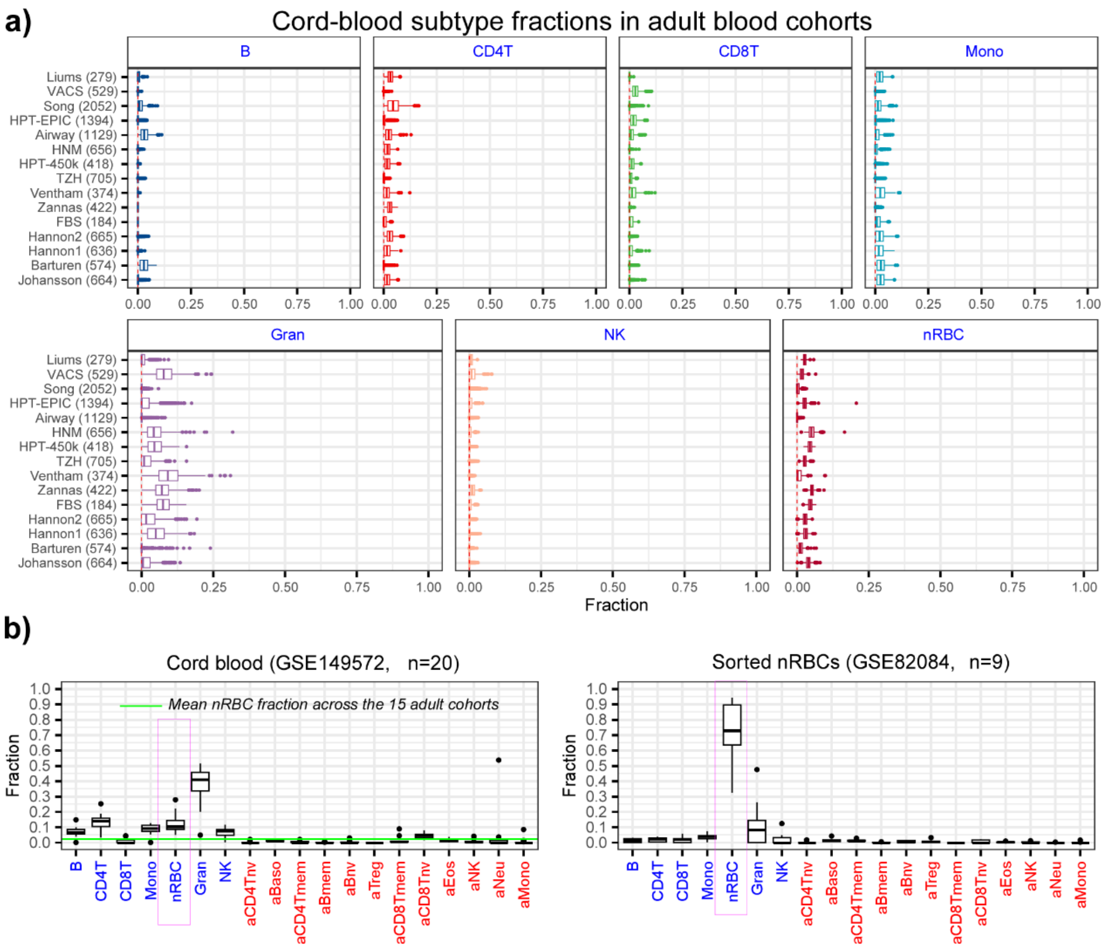

SI fig.S2: Small but non-negligible fractions of cord-blood (i.e youthful) cell types in adult cohorts. a) Boxplots of estimated cord-blood granulocyte, monocyte, natural killer (NK), B- cell, CD4+ T cell, CD8+ T cell and nucleated red blood cell (nRBC) fractions (x-axis) in each of 15 adult blood cohorts (y-axis). The sample size for each cohort is provided (y-axis). **b)** Boxplots of the estimated 19 immune cell-type fractions (from UniLIFE panel) in 20 cord blood samples (Left) and 9 sorted nRBCs (Right). Cord-blood cell subtypes are labeled in blue, adult blood cell-types in red. The horizontal green line indicates the mean nRBC fraction as computed across the 15 adult cohorts in panel a).

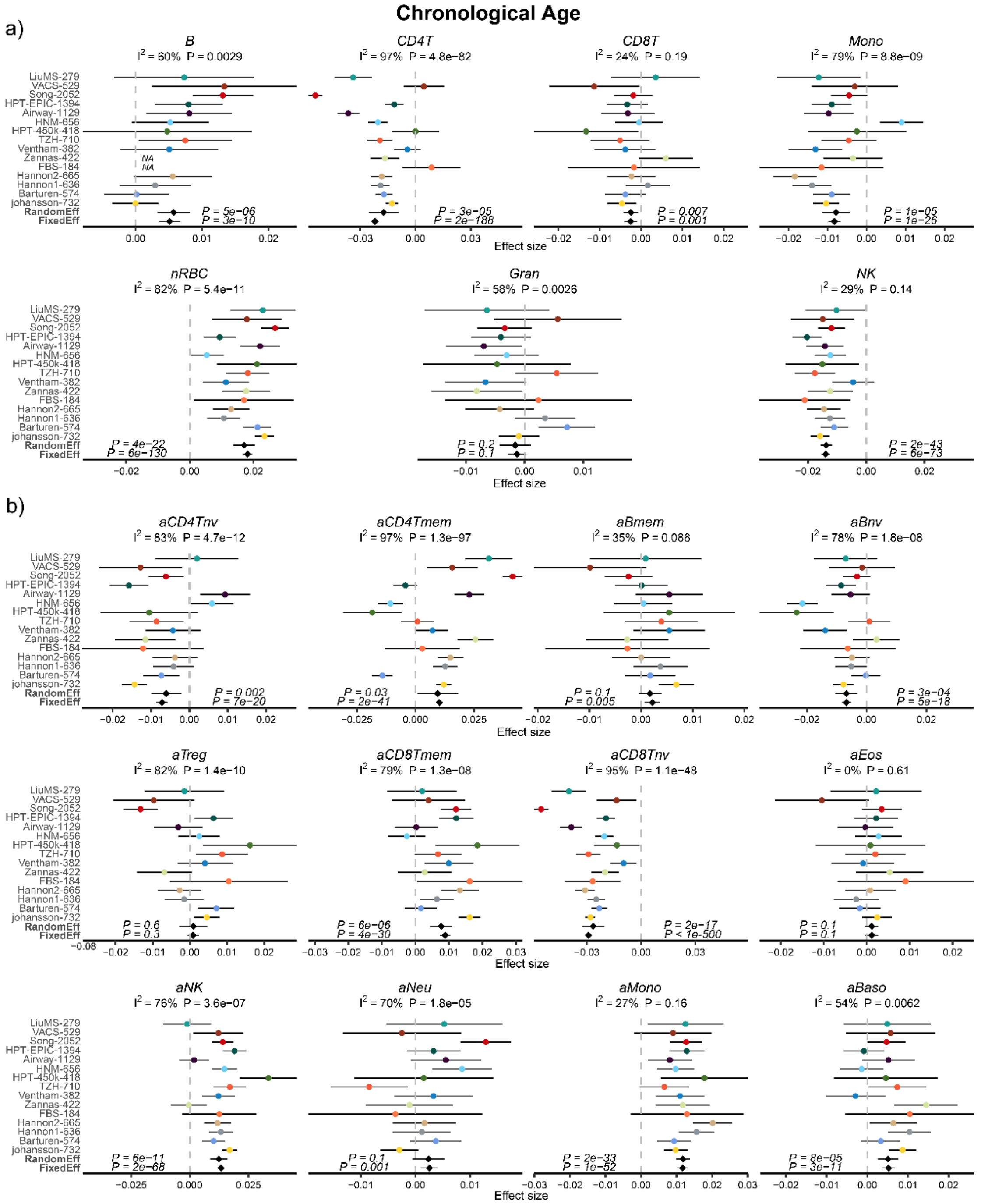

SI fig.S3: Forest plot of associations of UniLIFE IC type fractions with age. a) Forest plots of association for 7 youthful (cord-blood) immune cell-type fractions with chronological age (adjusting for sex), in each of 15 adult whole blood cohorts. Number of samples in each cohort is given. P-values of a random effect (RE) and fixed effect model (FE) are given. I^2 heterogeneity index and P-value is given above each panel. **b)** As a), but for the 12 adult immune cell-types.

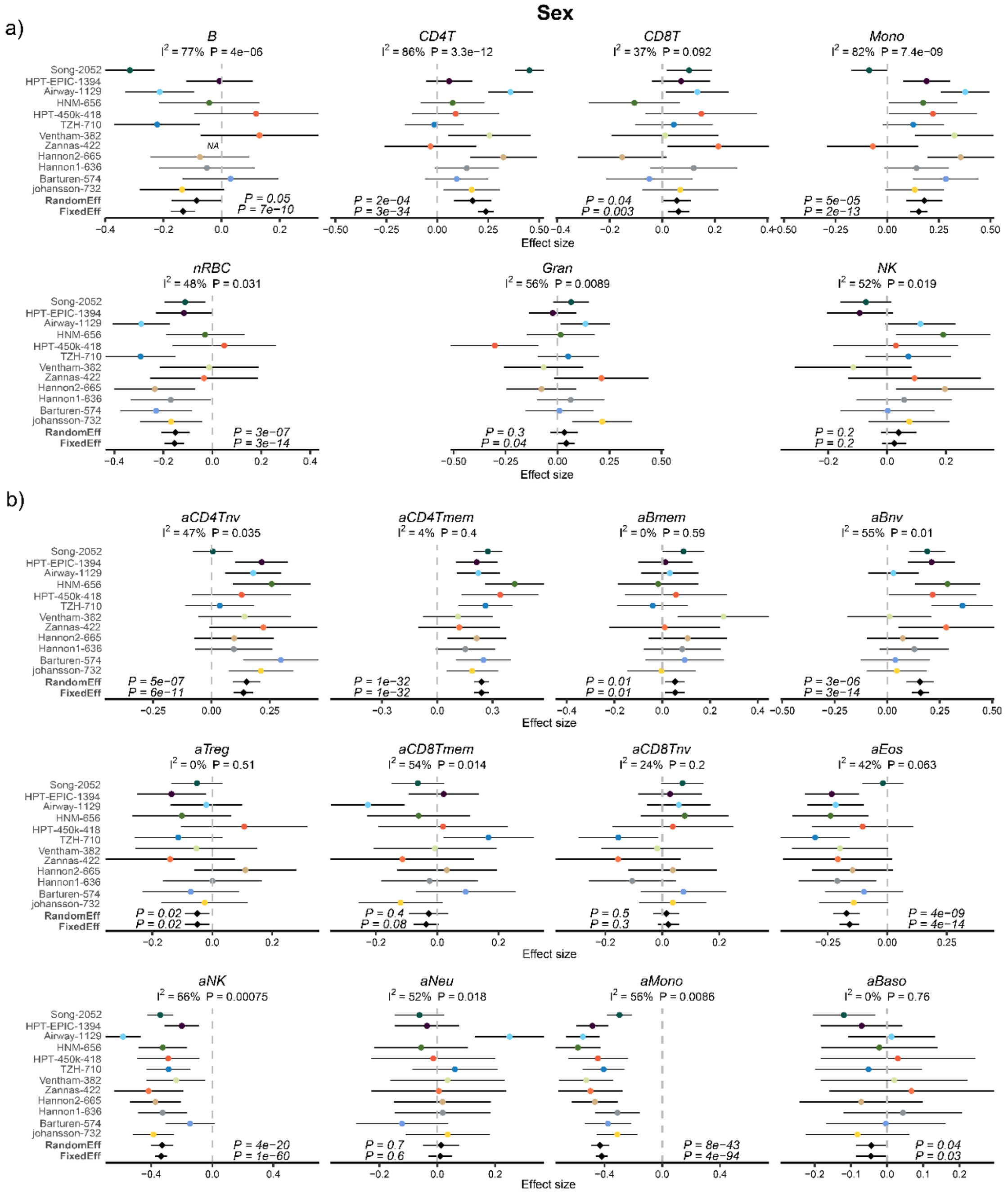

SI fig.S4: Forest plot of associations of UniLIFE IC type fractions with sex. a) Forest plots of association for 7 youthful (cord-blood) immune cell-type fractions with sex (adjusting for sage), in each of 15 adult whole blood cohorts. Positive effect sizes mean higher fractions in women compared to men. Number of samples in each cohort is given. P-values of a random effect (RE) and fixed effect model (FE) are given. I^2 heterogeneity index and P-value is given above each panel. **b)** As a), but for the 12 adult immune cell-types.

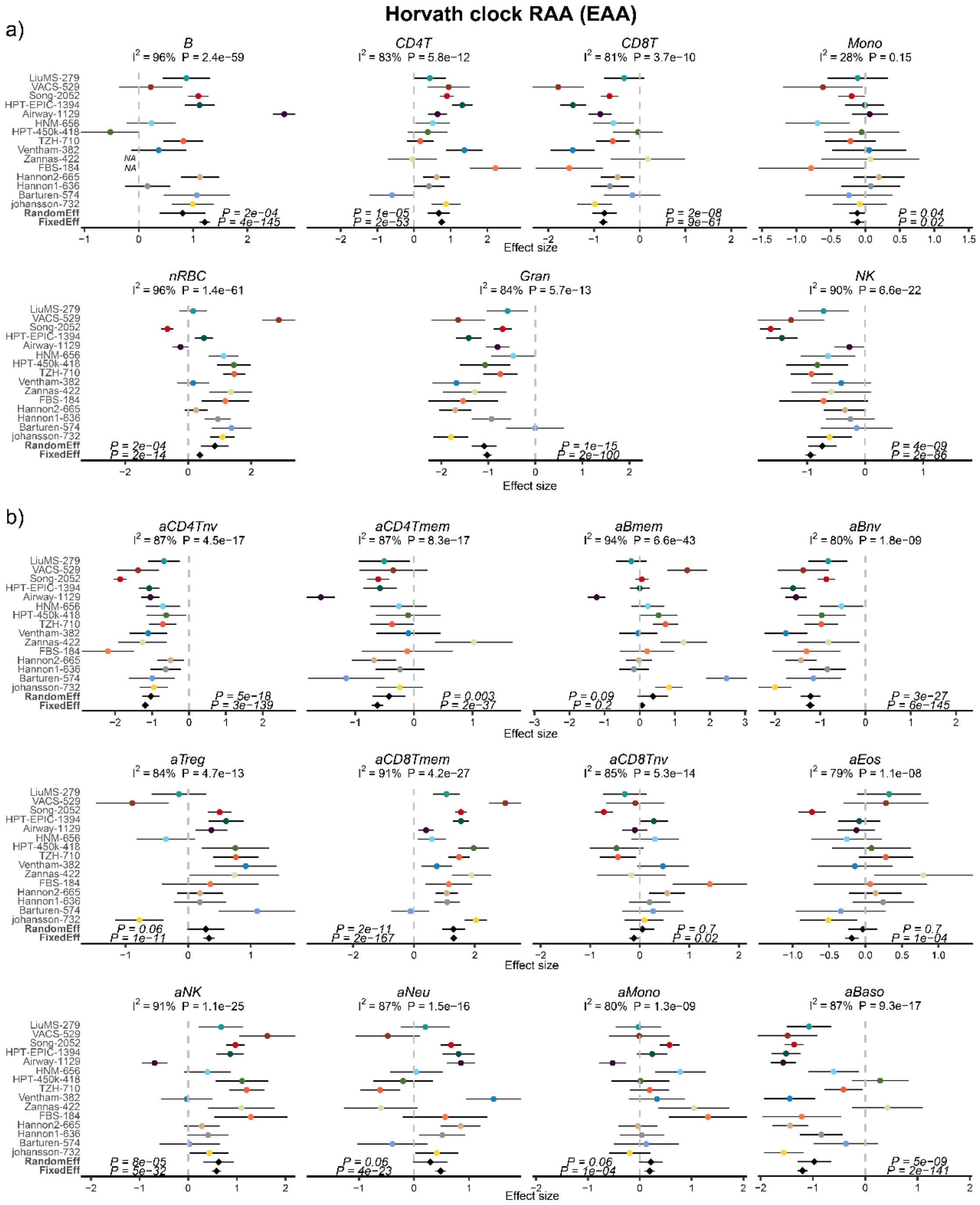

SI fig.S5: Forest plot of associations between Horvath clock and cell-type fractions. **a)** Forest plots of association for 7 youthful (cord-blood) immune cell-type fractions with the relative age acceleration (RAA) from Horvath's clock, in each of 15 adult whole blood cohorts. Number of samples in each cohort is given. P-values of a random effect (RE) and fixed effect model (FE) are given. I^2 heterogeneity index and P-value is given above each panel. **b)** As a), but for the 12 adult immune cell-types.

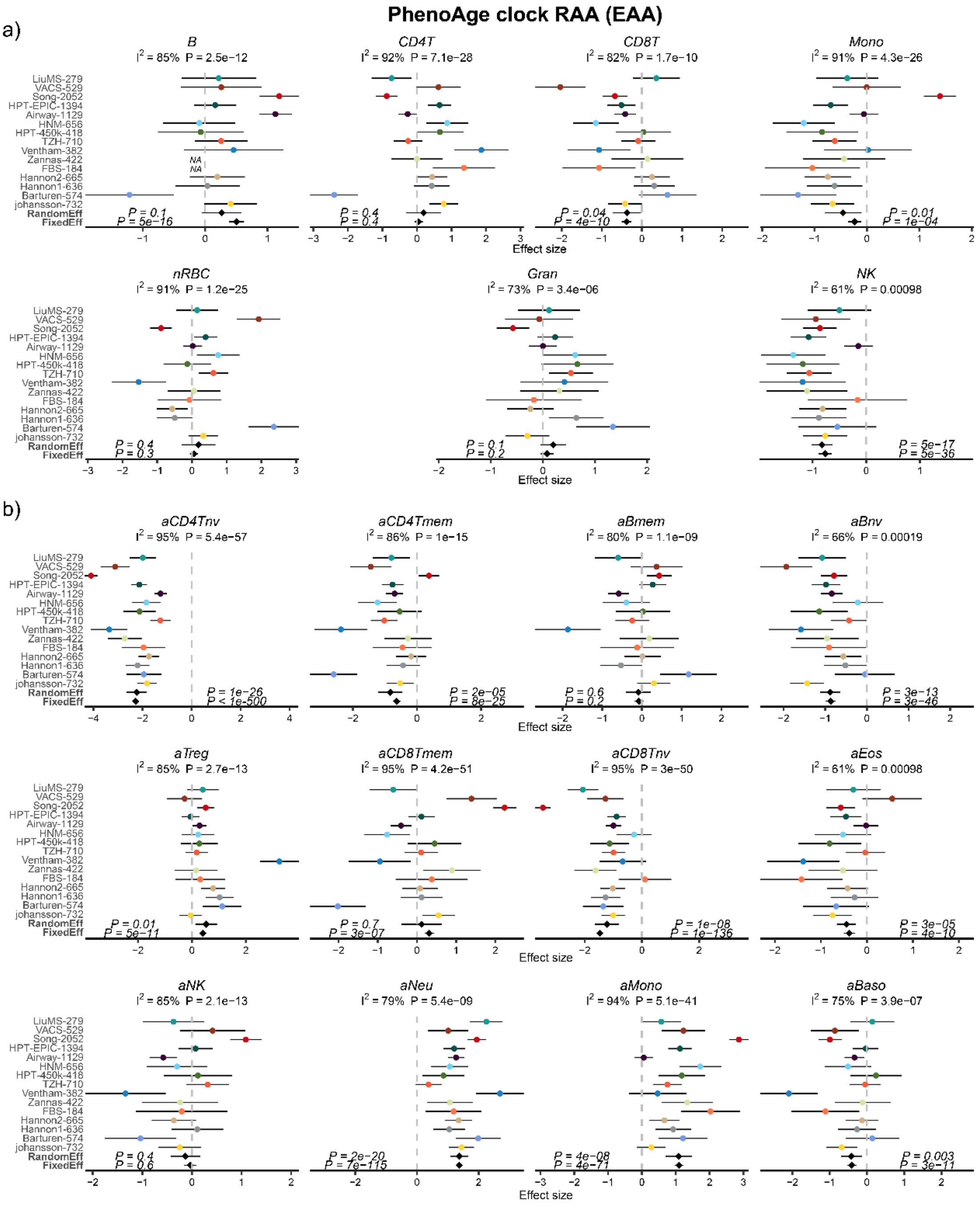

SI fig.S6: Forest plot of associations between PhenoAge and cell-type fractions. a) Forest plots of association for 7 youthful (cord-blood) immune cell-type fractions with the relative age acceleration (RAA) from PhenoAge clock, in each of 15 adult whole blood cohorts. Number of samples in each cohort is given. P-values of a random effect (RE) and fixed effect model (FE) are given. I^2 heterogeneity index and P-value is given above each panel. **b)** As a), but for the 12 adult immune cell-types.

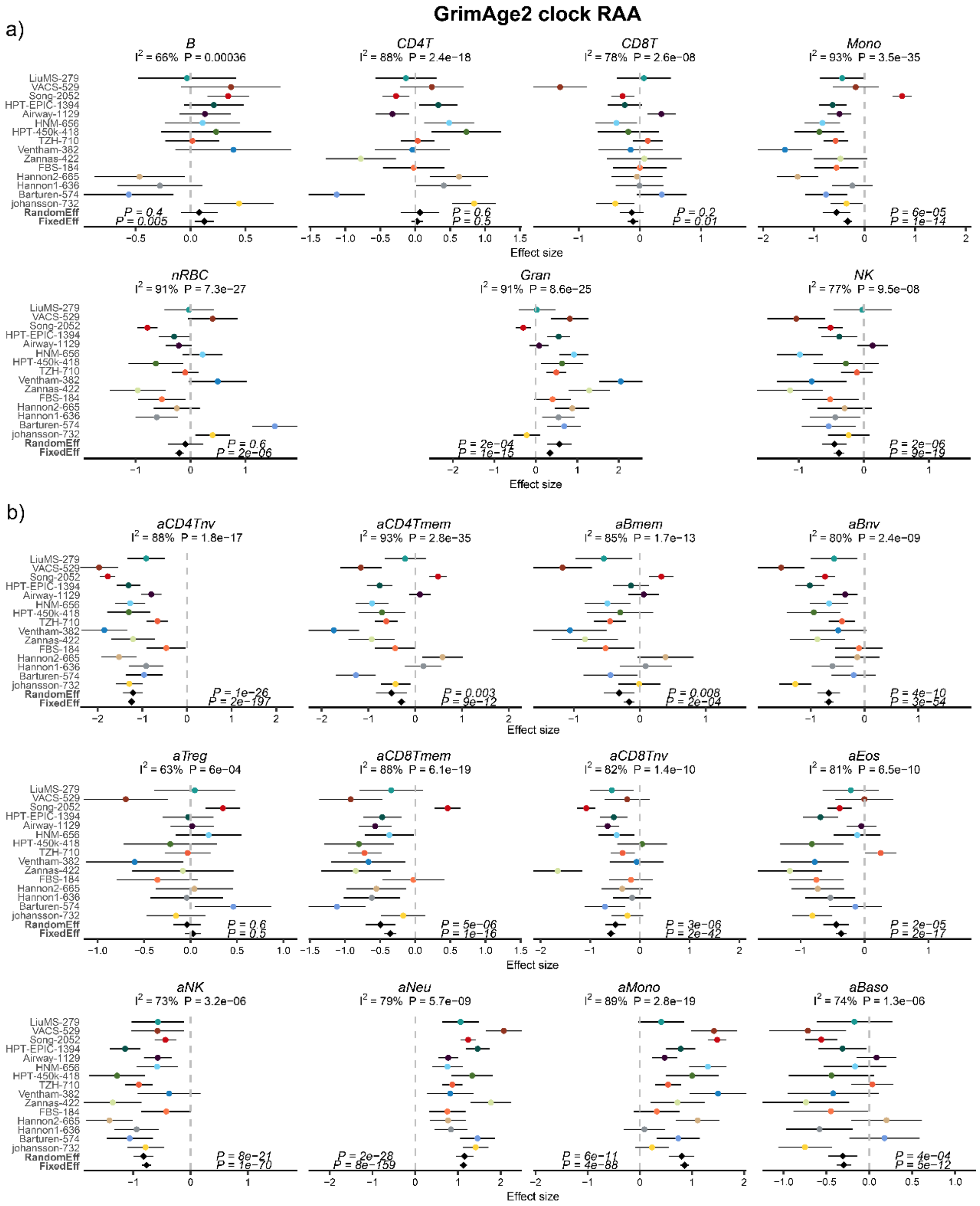

SI fig.S7: Forest plot of associations between GrimAge2 and cell-type fractions. a) Forest plots of association for 7 youthful (cord-blood) immune cell-type fractions with the relative age acceleration (RAA) from GrimAge2 clock, in each of 15 adult whole blood cohorts. Number of samples in each cohort is given. P-values of a random effect (RE) and fixed effect model (FE) are given. I^2 heterogeneity index and P-value is given above each panel. **b)** As a), but for the 12 adult immune cell-types.

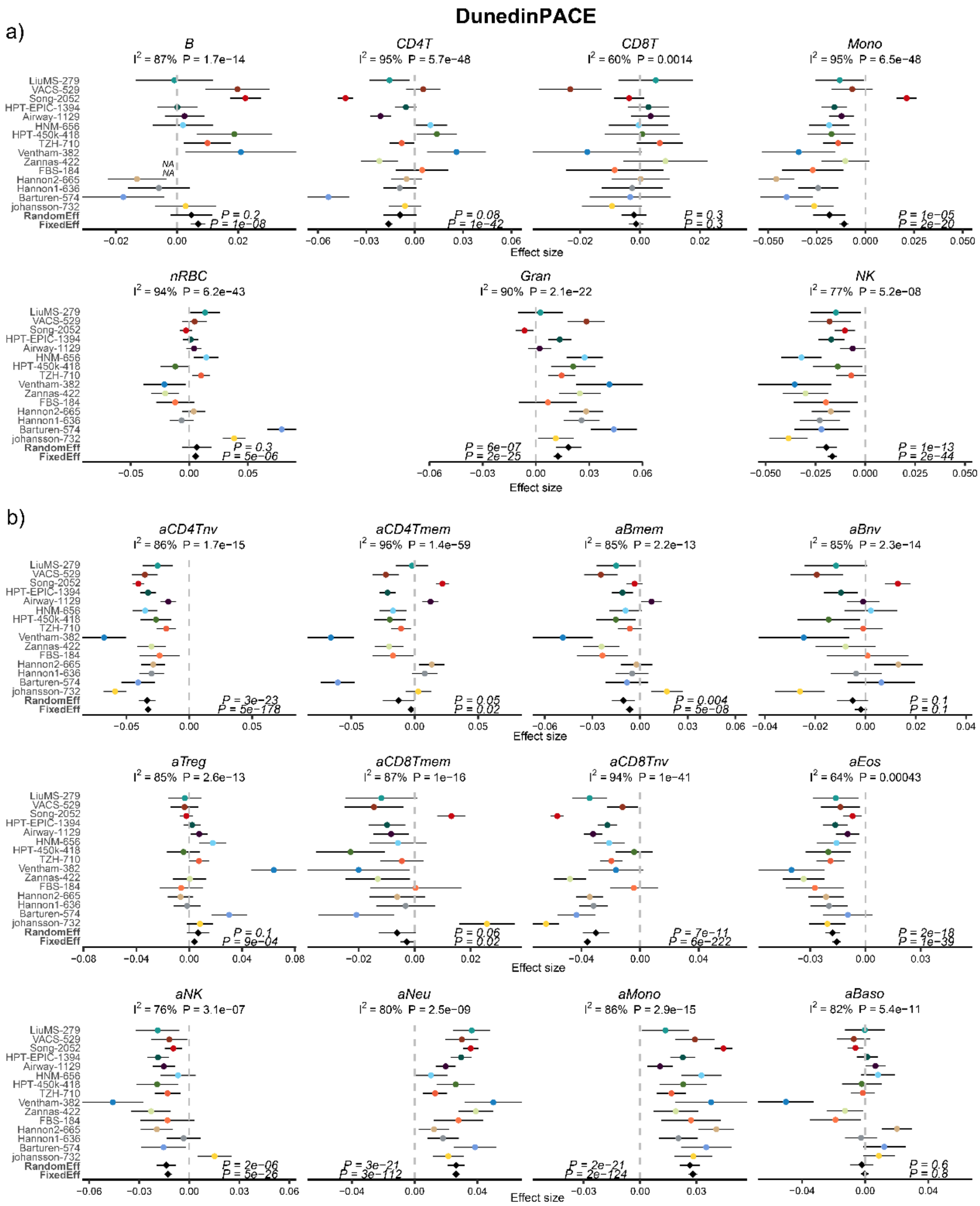

SI fig.S8: Forest plot of associations between DunedinPACE and cell-type fractions. a) Forest plots of association for 7 youthful (cord-blood) immune cell-type fractions with DunedinPACE of aging, in each of 15 adult whole blood cohorts. Number of samples in each cohort is given. P-values of a random effect (RE) and fixed effect model (FE) are given. I^2 heterogeneity index and P-value is given above each panel. **b)** As a), but for the 12 adult immune cell-types.

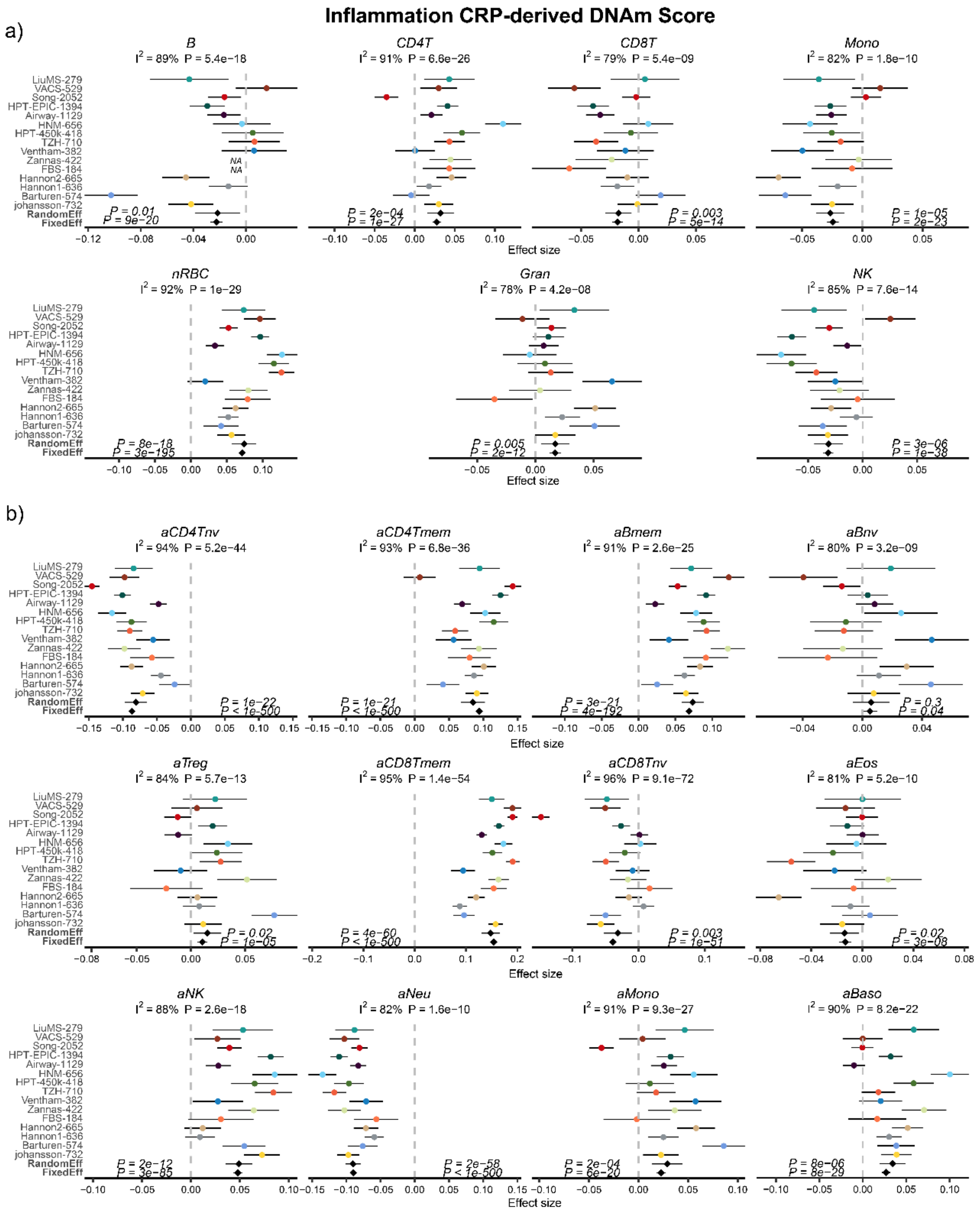

SI fig.S9: Forest plot of associations between inflammaging and cell-type fractions. **a)** Forest plots of association for 7 youthful (cord-blood) immune cell-type fractions with the CRP-derived DNAm-based inflammaging score in each of 15 adult whole blood cohorts. Number of samples in each cohort is given. P-values of a random effect (RE) and fixed effect model (FE) are given. I^2 heterogeneity index and P-value is given above each panel. **b)** As a), but for the 12 adult immune cell-types.

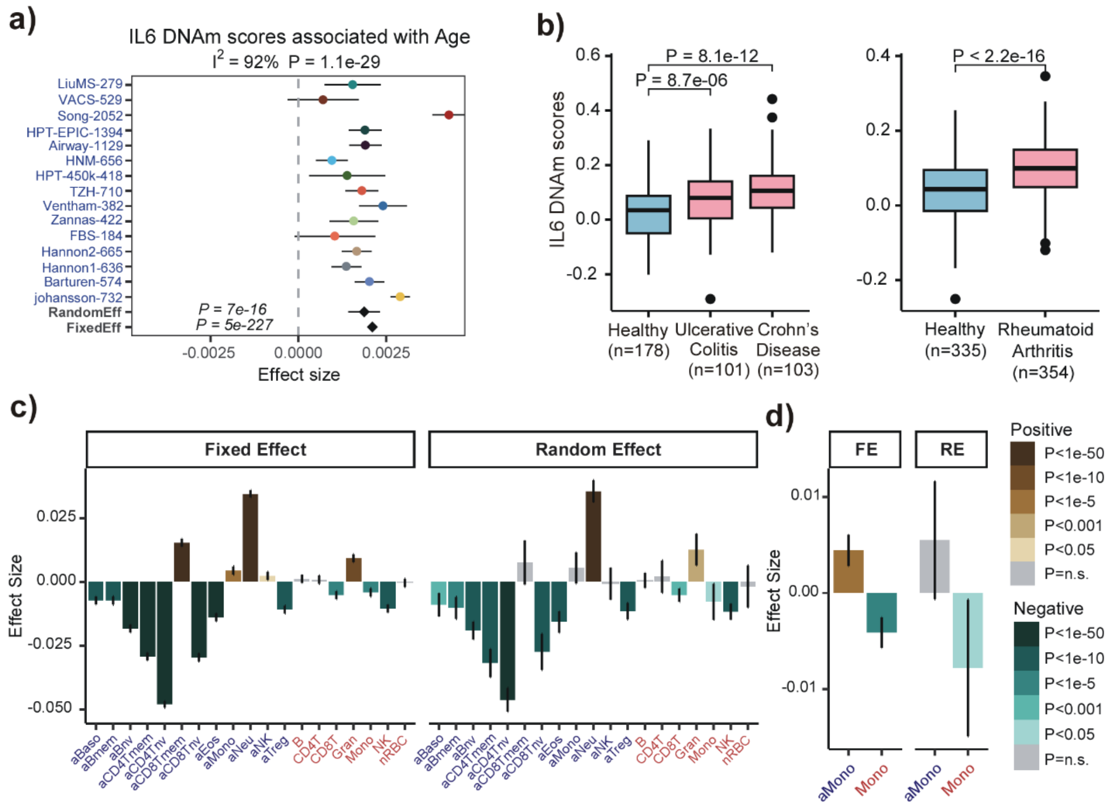

SI fig.S10: Association of IL6 with 19 immune cell fractions. a) Forest plot of association of a DNAm-based IL6 score with chronological age, as assessed over a meta-analysis of 15 whole blood cohorts. P-value of a random and fixed effect meta-analysis model is given. Error bars represent 95% confidence intervals. The I^2 heterogeneity index and P-value is given above panel. **b)** Boxplots displaying the IL6 DNAm-score in two DNAm datasets profiling healthy individuals and people with inflammatory conditions, as indicated. P-values are from a one-tailed Wilcoxon rank sum test. **c)** Barplots with 95% confidence intervals displaying the association of the DNAm-based IL6 score with 19 immune cell-type fractions, under a fixed and random effect models. **d)** As c), but only displaying the adult monocyte (aMono) and young monocyte (Mono) fractions.

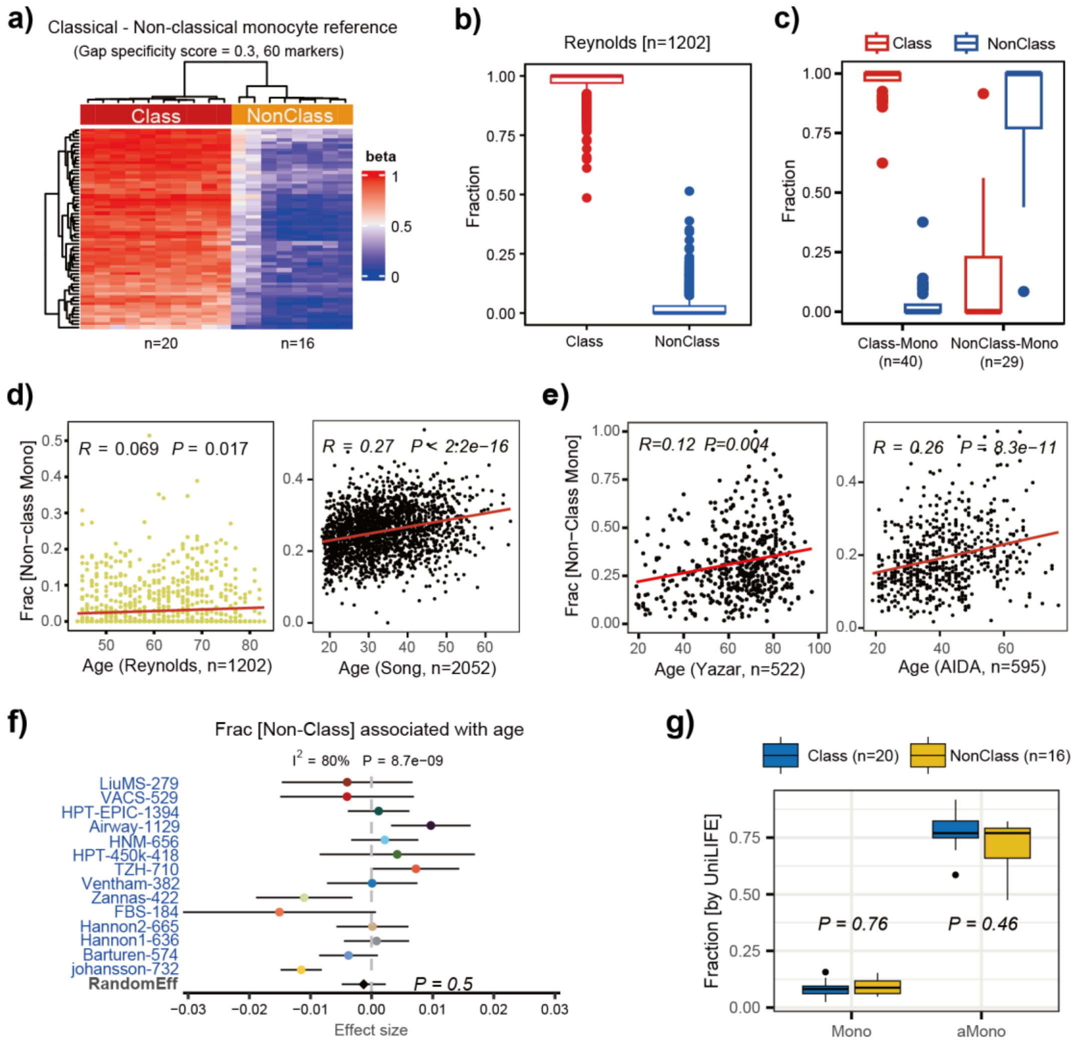

SI fig.S11: Non-classical monocyte fraction increases with age but is distinct from young-adult monocyte subtyping. a) DNAm beta-value heatmap of 60 marker CpGs with gap-specificity score > 0.3, discriminating 20 classical from 16 non-classical sorted monocyte samples. **b)** Validation of the monocyte DNAm reference panel of a) in 1202 sorted CD14+/CD16- (classical) monocyte samples from the MESA study. **c)** Validation of the monocyte DNAm reference panel in an independent DNAm dataset of classical and non-classical monocytes from SLE patients. **d) Left:** Scatterplot of the estimated non-classical monocyte fraction vs age in the MESA study. R-value and two-tailed P-value from linear regression is given. **Right:** Scatterplot displaying the estimated non-classical monocyte fraction vs chronological age in a PBMC DNAm dataset from Song et al. R-value and two-tailed P-value from a linear regression are given. **e)** Scatterplot of the fraction of non-classical monocytes vs age in the Yazar et al and AIDA scRNA-sequencing studies. Fractions were adjusted for pool (Yazar) and sex (Yazar and AIDA) when correlating to age and are shown for donors with a sufficient numbers of total monocyte cell counts (>50). Two-tailed P-value from linear regression are given. Cells were annotated into classical CD14+ and non-classical CD16+ subtypes using the single-cell annotations of Yazar et al and AIDA. **f)** Forest plot of association of the estimated non-classical monocyte fraction with age across whole

blood cohorts. **g)** Boxplots displaying the estimated adult (aMono) and young (Mono) monocyte fractions in the sorted classical and non-classical monocyte samples. P-values are from a two-tailed Wilcoxon test.

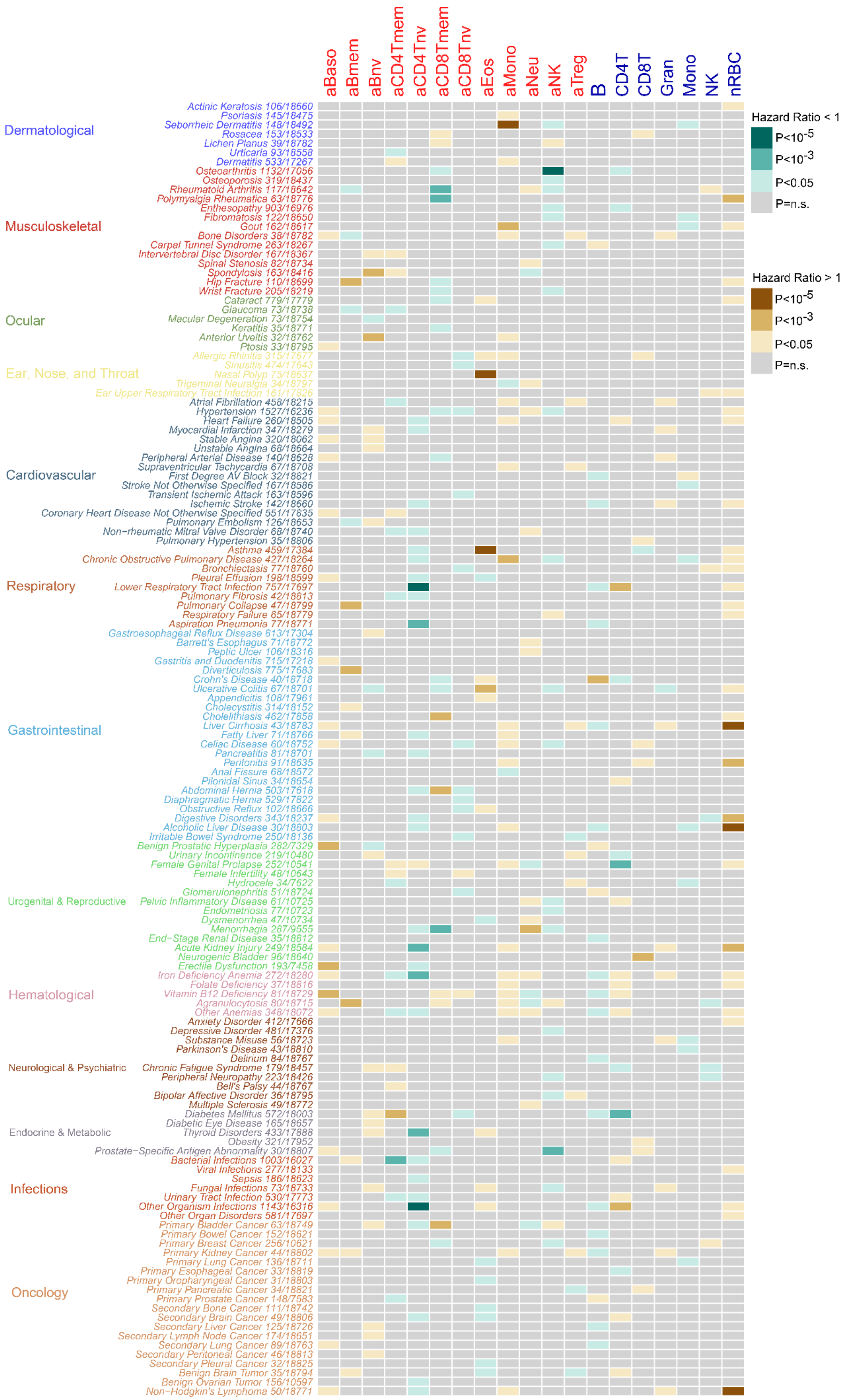
aBaso
aBmem
aBnv
aCD4Tmem
aCD4Tnv
aCD8Tmem
aCD8Tnv
aEos
aMono
aNeu
aNK
aTreg
B
CD4T
CD8T
Gran
Mono
NK
nRBC
Dermatological
Actinic Keratosis 106/18660
Psoriasis 145/18475
Seborrheic Dermatitis 148/18492
Rosacea 153/18533
Lichen Planus 39/18782
Urticaria 93/18558
Dermatitis 533/17267
Musculoskeletal
Osteoarthritis 1132/17056
Osteoporosis 319/18437
Rheumatoid Arthritis 117/18642
Polymyalgia Rheumatica 63/18776
Enthesopathy 903/16976
Fibromatosis 122/18650
Gout 162/18617
Bone Disorders 38/18782
Carpal Tunnel Syndrome 263/18267
Intervertebral Disc Disorder 167/18367
Spinal Stenosis 82/18734
Spondylosis 163/18416
Hip Fracture 110/18699
Wrist Fracture 205/18219
Ocular
Cataract 779/17779
Glaucoma 73/18738
Macular Degeneration 73/18754
Keratitis 35/18771
Anterior Uveitis 32/18762
Ptosis 33/18795
Ear, Nose, and Throat
Allergic Rhinitis 315/17577
Sinusitis 474/17643
Nasal Polyp 75/18637
Trigeminal Neuralgia 34/18797
Ear Upper Respiratory Tract Infection 161/17826
Cardiovascular
Atrial Fibrillation 458/18215
Hypertension 1527/16236
Heart Failure 260/18505
Myocardial Infarction 347/18279
Stable Angina 320/18062
Unstable Angina 68/18664
Peripheral Arterial Disease 140/18628
Supraventricular Tachycardia 67/18708
First Degree AV Block 32/18821
Stroke Not Otherwise Specified 167/18586
Transient Ischemic Attack 163/18596
Ischemic Stroke 142/18660
Coronary Heart Disease Not Otherwise Specified 551/17835
Pulmonary Embolism 126/18653
Non−rheumatic Mitral Valve Disorder 68/18740
Pulmonary Hypertension 35/18806
Respiratory
Asthma 459/17384
Chronic Obstructive Pulmonary Disease 427/18264
Bronchiectasis 77/18760
Pleural Effusion 198/18599
Lower Respiratory Tract Infection 757/17697
Pulmonary Fibrosis 42/18813
Pulmonary Collapse 47/18799
Respiratory Failure 65/18779
Aspiration Pneumonia 77/18771
Gastrointestinal
Gastroesophageal Reflux Disease 813/17304
Barrett's Esophagus 71/18772
Peptic Ulcer 106/18316
Gastritis and Duodenitis 715/17218
Diverticulosis 775/17683
Crohn's Disease 40/18718
Ulcerative Colitis 67/18701
Appendicitis 108/17961
Cholecystitis 314/18152
Cholelithiasis 462/17858
Liver Cirrhosis 43/18783
Fatty Liver 71/18766
Celiac Disease 60/18752
Pancreatitis 81/18701
Peritonitis 91/18635
Anal Fissure 68/18572
Pilonidal Sinus 34/18654
Abdominal Hernia 503/17616
Diaphragmatic Hernia 529/17822
Obstructive Reflux 102/18666
Digestive Disorders 343/18237
Alcoholic Liver Disease 30/18803
Irritable Bowel Syndrome 250/18136
Urogenital & Reproductive
Benign Prostatic Hyperplasia 282/7329
Urinary Incontinence 219/10480
Female Genital Prolapse 252/10541
Female Infertility 48/10643
Hydrocele 34/7622
Glomerulonephritis 51/18724
Pelvic Inflammatory Disease 61/10725
Endometriosis 77/10723
Dysmenorrhea 47/10734
Menorrhagia 287/9555
End−Stage Renal Disease 35/18812
Acute Kidney Injury 249/18584
Neurogenic Bladder 96/18640
Erectile Dysfunction 193/7458
Hematological
Iron Deficiency Anemia 272/18280
Folate Deficiency 37/18816
Vitamin B12 Deficiency 81/18729
Agranulocytosis 80/18715
Other Anemias 348/18072
Neurological & Psychiatric
Anxiety Disorder 412/17666
Depressive Disorder 481/17376
Substance Misuse 56/18723
Parkinson's Disease 43/18810
Delirium 84/18767
Chronic Fatigue Syndrome 179/18457
Peripheral Neuropathy 223/18426
Bell's Palsy 44/18767
Bipolar Affective Disorder 36/18795
Multiple Sclerosis 49/18772
Endocrine & Metabolic
Diabetes Mellitus 572/18003
Diabetic Eye Disease 165/18657
Thyroid Disorders 433/17888
Obesity 321/17952
Prostate−Specific Antigen Abnormality 30/18807
Infections
Bacterial Infections 1003/16027
Viral Infections 277/18133
Sepsis 186/18623
Fungal Infections 73/18733
Urinary Tract Infection 530/17773
Other Organism Infections 1143/16316
Other Organ Disorders 581/17697
Oncology
Primary Bladder Cancer 63/18749
Primary Bowel Cancer 152/18621
Primary Breast Cancer 256/10621
Primary Kidney Cancer 44/18802
Primary Lung Cancer 136/18711
Primary Esophageal Cancer 33/18819
Primary Oropharyngeal Cancer 31/18803
Primary Pancreatic Cancer 34/18821
Primary Prostate Cancer 148/7583
Secondary Bone Cancer 111/18742
Secondary Brain Cancer 49/18806
Secondary Liver Cancer 125/18726
Secondary Lymph Node Cancer 174/18651
Secondary Lung Cancer 89/18763
Secondary Peritoneal Cancer 46/18813
Secondary Pleural Cancer 32/18825
Benign Brain Tumor 35/18794
Benign Ovarian Tumor 156/10597
Non−Hodgkin's Lymphoma 50/18771
Hazard Ratio < 1
P<10^-5
P<10^-3
P<0.05
P=n.s.
Hazard Ratio > 1
P<10^-5
P<10^-3
P<0.05
P=n.s.

SI fig.S12: Associations between 19 immune cell type fractions and medical conditions in GenS cohort. The heatmap illustrates the associations between the UniLIFE 19 immune cell type fractions (7 youthful (cord-blood) as well as 12 adult immune cell types) and a wide range of medical conditions, divided into 13 groups based on the primary human systems and organs they impact. Each row represents a distinct condition, while columns correspond to the 19 cell subtypes (listed at the top). The numbers after the term indicate the number of individuals with the medical condition and the total number of individuals with or without the condition. The entries in the heatmap are color-coded to indicate hazard ratio (HR) significance levels and directions: For HR greater than 1, which indicates an increased risk of the disease, the colors transition towards dark brown. For HR less than 1, which indicates a decreased risk of the disease, the colors transition towards dark green.

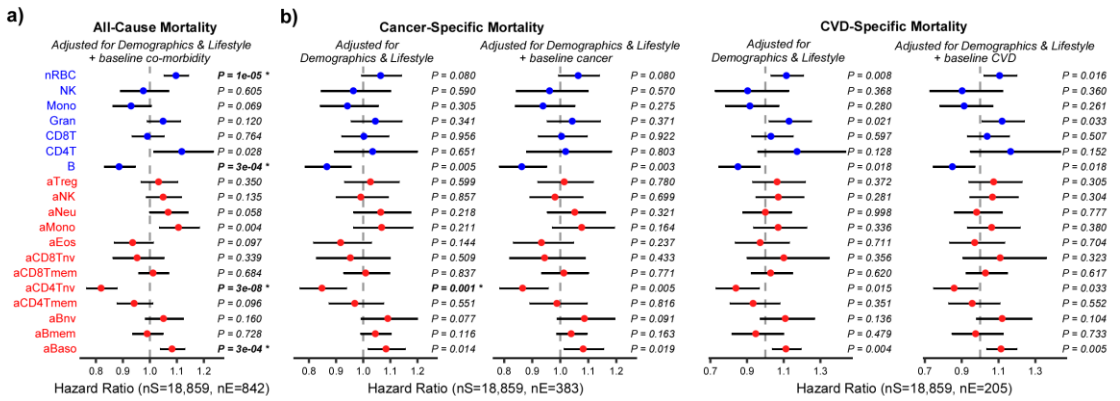

SI fig.S13: Associations of 19 immune cell type fractions with all-cause and disease-specific mortality in GenS cohort, adjusting for baseline co-morbidity. a) Forest plots depicting the association of the 19 immune-cell type fractions with all-cause mortality in the GenS cohort after adjusting for all demographic and lifestyle risk factors, as well as baseline co-morbidity (defined as cancer, CVD, T2D, COPD or depression). The x-axis labels the Hazard Ratio (HR) and 95% confidence interval is given for each estimate. Left panel is adjusted for age, sex, race and baseline co-morbidity.. Number of samples (nS) and death events (nE) is given. **b)** As a) but for cancer and cardiovascular disease (CVD) specific mortality. * Associations significant under Bonferroni adjustment for multiple testing.

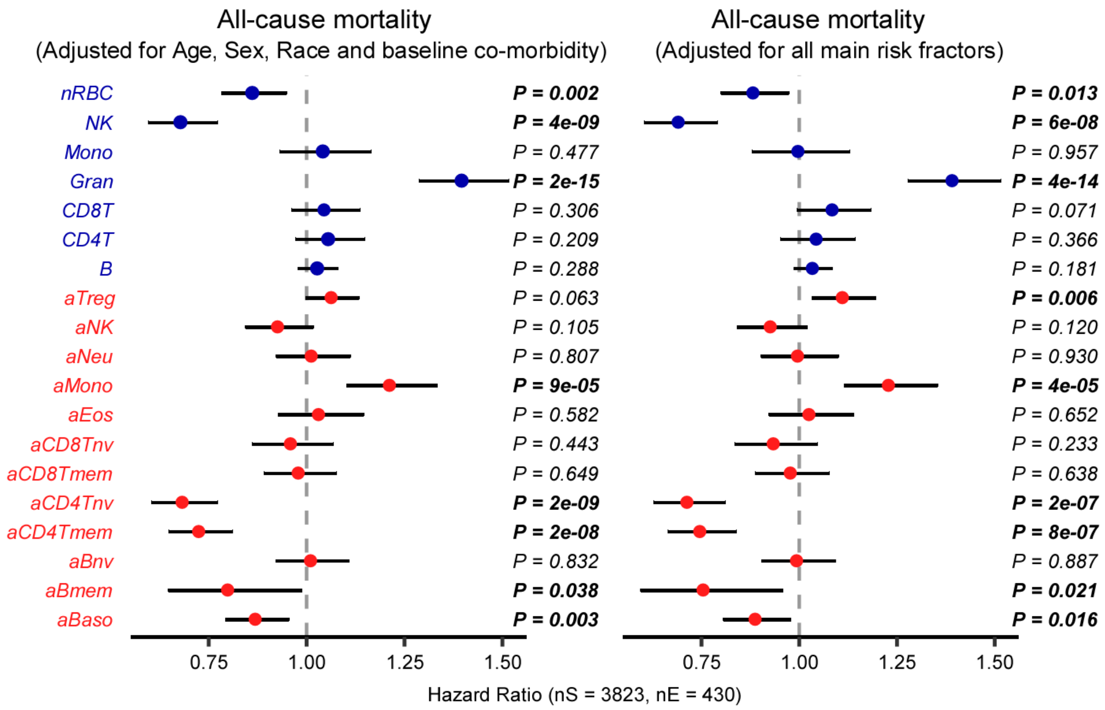

SI fig.S14: Associations between 19 immune cell type fractions and all-cause mortality in the MGBB cohort. Forest plots depicting the association of the 19 immune-cell type fractions with all-cause mortality in the MGBB cohort. The x-axis labels the Hazard Ratio (HR) and 95% confidence interval is given for each estimate. Left panel is adjusted for age, sex, race and baseline co-morbidity. Right panel is adjusted for these factors as well as for smoking, BMI and alcohol consumption. Number of samples (nS) and death events (nE) is given below x-axis.

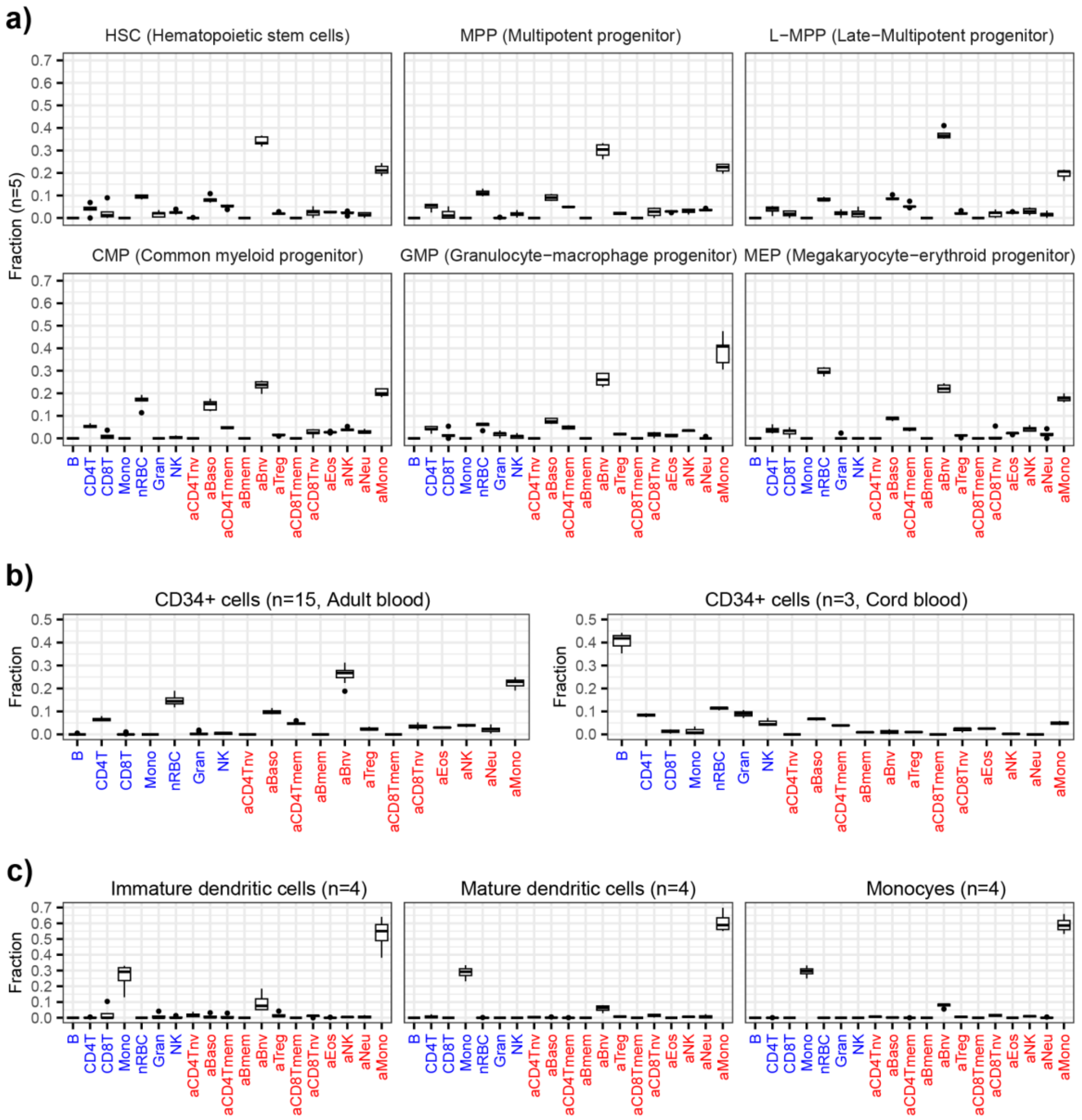

SI fig.S15: UniLIFE's nRBC fraction is small in sorted dendritic cells and hematopoietic stem/progenitor cells. a) Boxplots show the estimated fractions (y-axis) of 19 immune cell types (x-axis) in sorted cells representing 6 hematopoietic progenitor cell types from the bone marrow of 5 donors, as estimated by UniLIFE. Blue represents cord blood IC types, while red represents adult IC types. **b)** as a), but for two sorted CD34+ cell DNAm datasets, one from adults (n=15) and another from cord-blood (n=3). **c)** as a), but for sorted dendritic cells and monocytes.

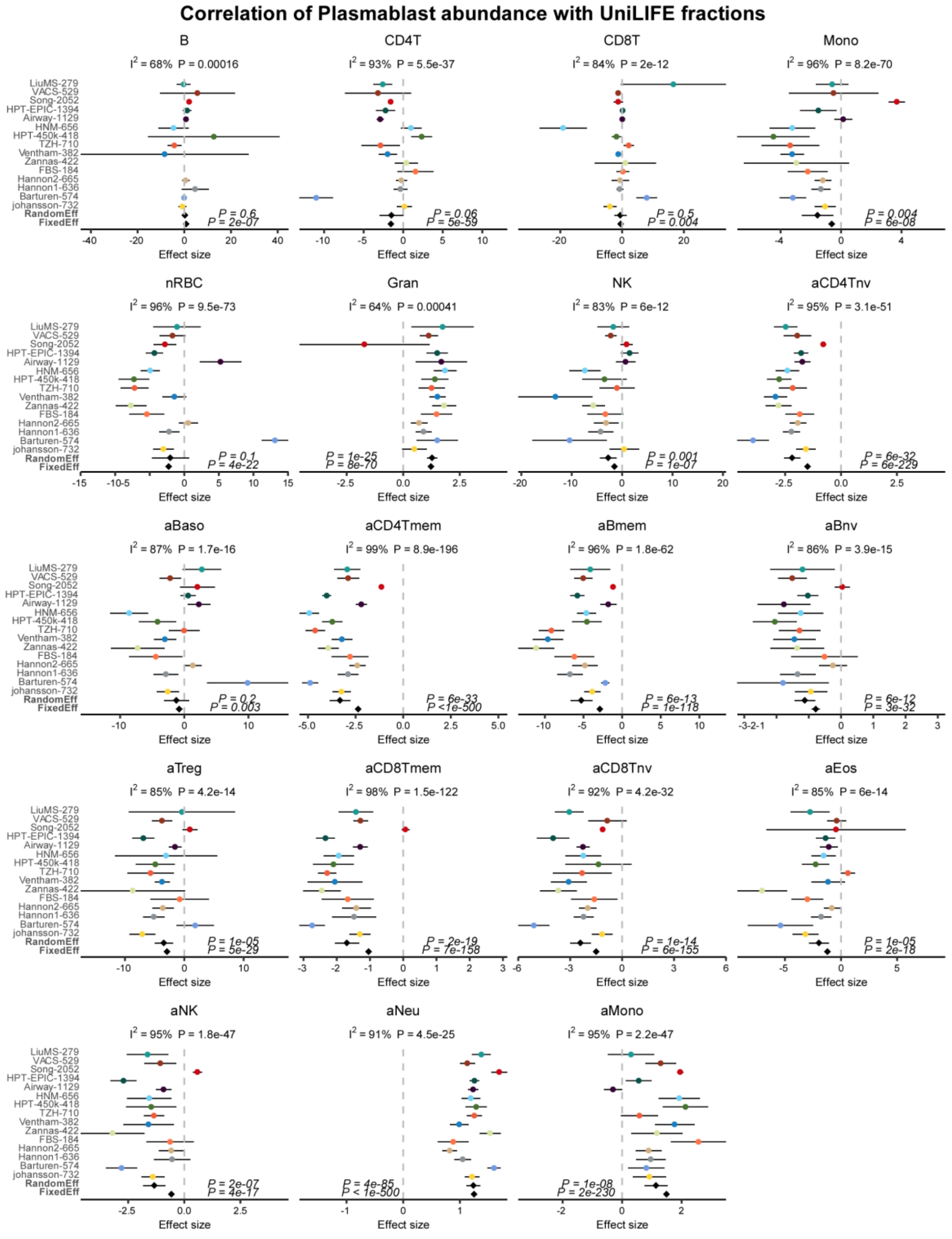

SI fig.S16: Forest plots of association of UniLIFE fractions with the plasmablast fraction estimator. Forest plots of association for the 19 UniLIFE fractions with the independent plasmablast fraction estimator, in each of 15 adult whole blood cohorts. Number of samples in each cohort is given. P-values of a random effect (RE) and fixed effect model (FE) are given. I2 heterogeneity index and P-value is given above each panel.

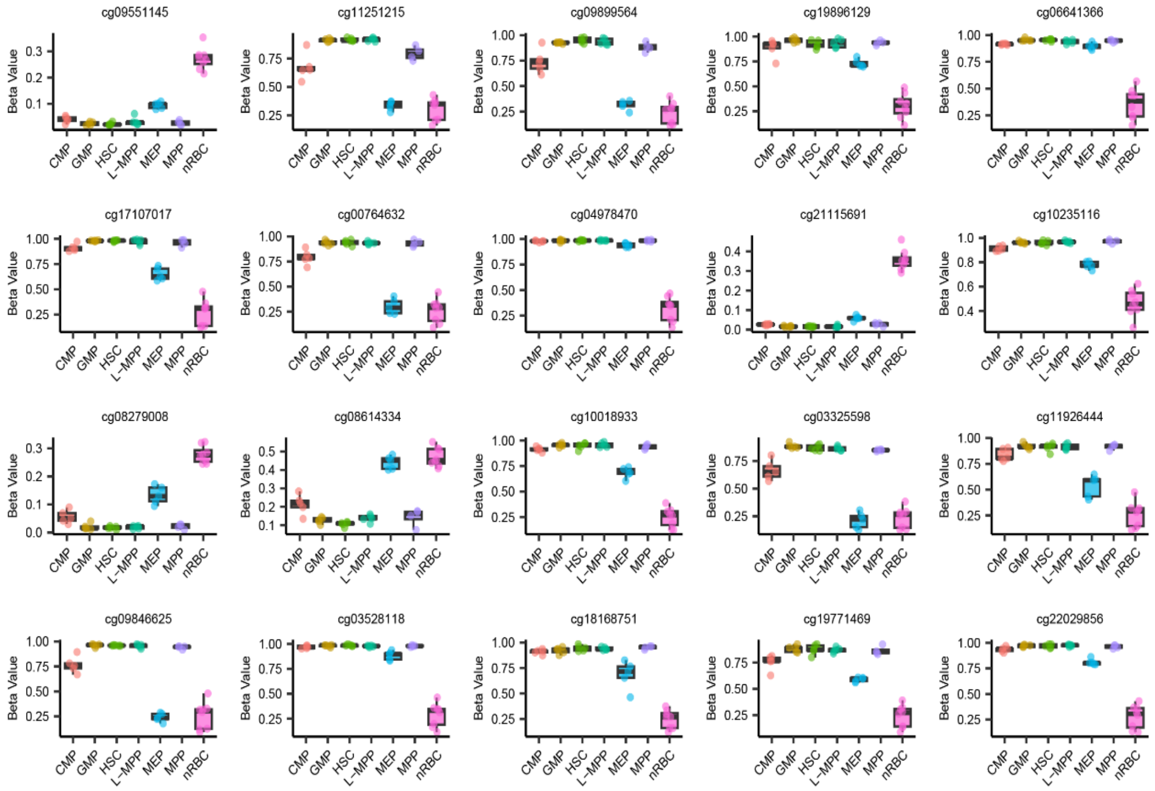

SI fig.S17: DNAm values of randomly selected UniLIFE nRBC markers across nRBCs and HPCs. Boxplots compare the DNAm-values of various HPCs from HM450k Jung et al (GSE63409) and nRBCs from HM450k Bakulski and de Goede et al, for 20 randomly selected nRBC markers from our UniLIFE panel. CMP=common myeloid progenitor (n=5), GMP=granulocyte-myeloid progenitor (n=5), HSC=hematopoietic stem-cell (n=5), L-MPP=lymphoid multipotent progenitor (n=5), MEP=megakaryocyte erythrocyte progenitor (n=5), MPP=multipotent progenitor (n=4), nRBC=nucleated red blood cells (n=8).

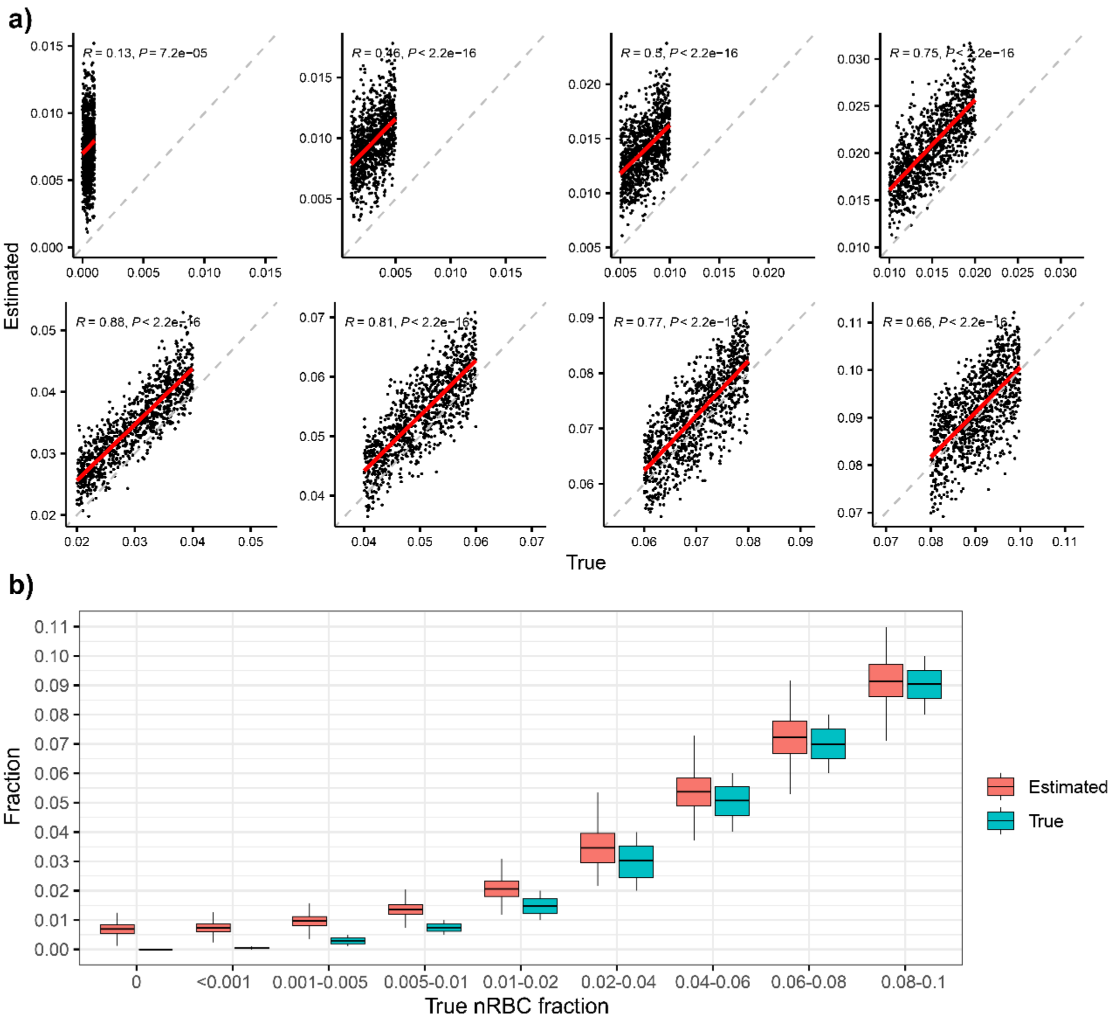

SI fig.S18: UniLIFE as applied to simulated in-silico mixtures containing a non-zero nRBC fraction. a) Scatterplot of the estimated nRBC fraction (y-axis) against the true nRBC fraction (x-axis) for variable spike-in proportions of nRBC cells. Each panel displays 1000 whole blood mixtures where the nRBC fraction is constrained to be in particular bins (<0.001, 0.001-0.005, 0.005-0.01, 0.01-0.02, 0.02-0.04, 0.04-0.06, 0.06-0.08, 0.08-0.1). R-value and P-value of a linear regression are given. **b)** Boxplots comparing the estimated and true nRBC fractions for the different bins, now also including the case where no nRBCs were spiked-in (labelled 0 on x-axis).

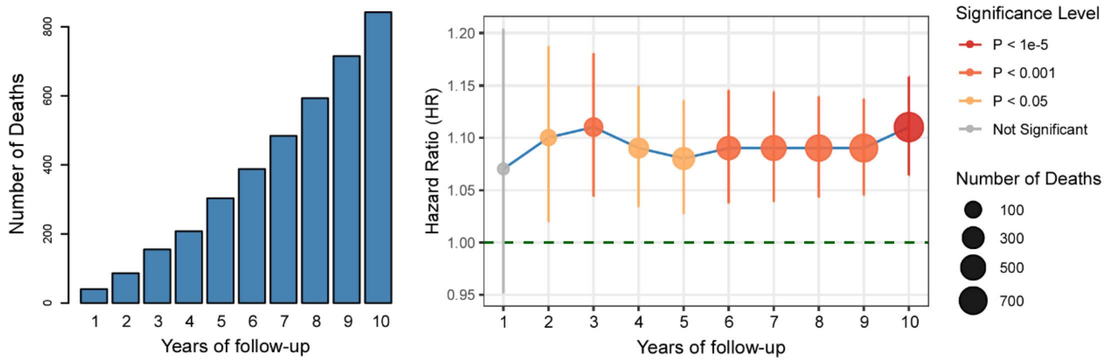

SI fig.S19: Association of nucleated red blood cell (nRBCs) fraction with all-cause mortality for varying number of follow-up years in GenS cohort. The histogram on the left shows the cumulative distribution of death events for 1 up to 10 years of follow-up after sample draw**.** The plot on the right shows the relationship between the fraction of nRBCs and risk of death for a number of follow-up years after the initial sample draw. The y-axis represents the Hazard Ratio (HR) of death, with a HR greater than 1 indicating that higher nRBC fraction increases risk of death. The x-axis shows the years of follow-up after sample draw.

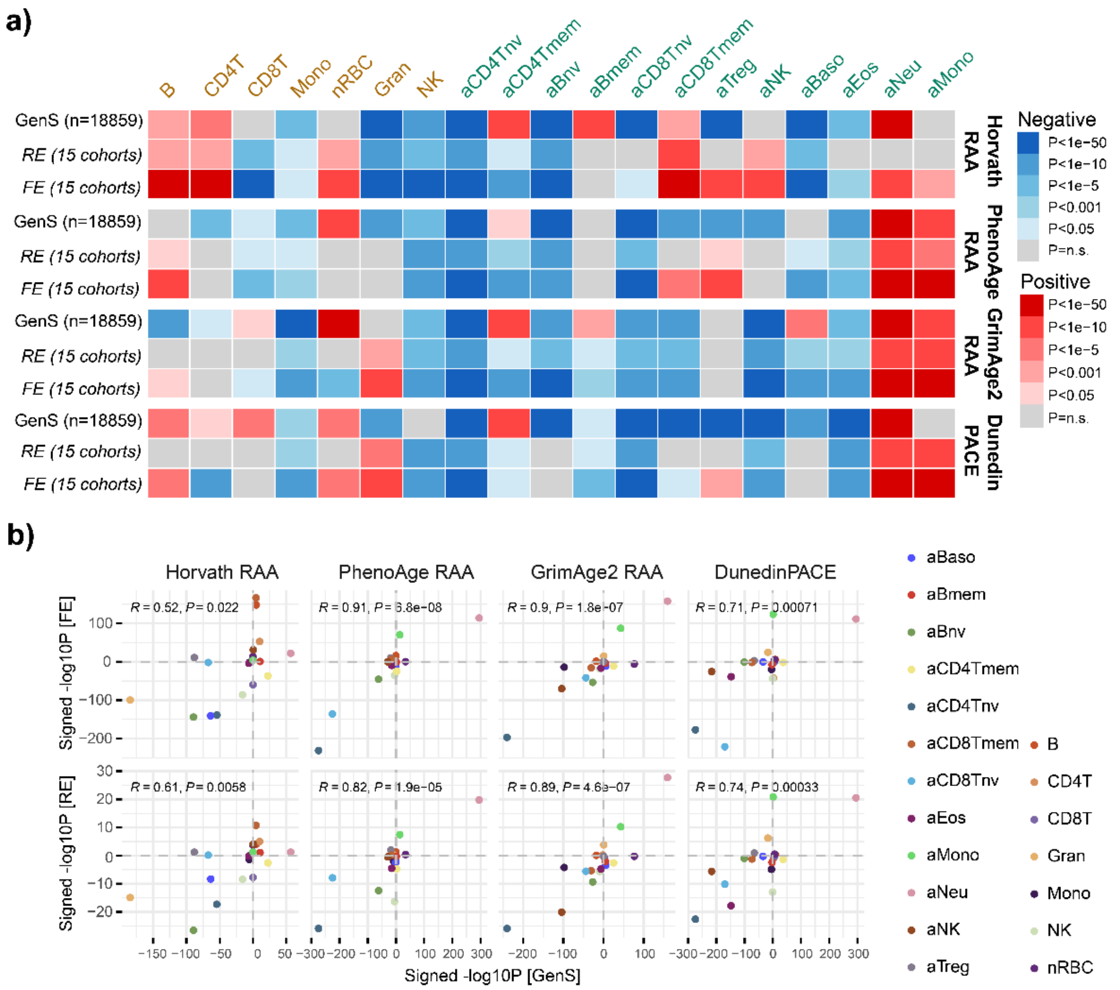

SI fig.S20: Association of epigenetic clock acceleration with 19 IC fractions in GenS cohort. a) Heatmaps of association between the relative age-acceleration (RAA for Horvath, PhenoAge and GrimAge2) or DunedinPACE with 19 immune cell-type fractions, as assessed over Generation Scotland (GenS, n=18859) or over a meta-analysis of 15 whole blood cohorts using fixed effect (FE) or random effect (RE) models. Heatmaps display the signed P-values. **b)** Scatterplots of signed P-values from the FE meta-analysis model vs GenS (top-panel) for each of the 4 clocks. Lower panel is the same but comparing GenS to the RE meta-analysis model. The signed P-values are the same as those shown in a). In each scatterplot panel, we display the R-value and P-value of a linear regression, assessing agreement between GenS and the meta-analysis.
